# The effect of loving-kindness meditation on positive emotions: a meta-analytic review

**DOI:** 10.3389/fpsyg.2015.01693

**Published:** 2015-11-03

**Authors:** Xianglong Zeng, Cleo P. K. Chiu, Rong Wang, Tian P. S. Oei, Freedom Y. K. Leung

**Affiliations:** ^1^Department of Psychology, The Chinese University of Hong KongHong Kong, China; ^2^Department of Sociology, Nanjing Forestry UniversityNanjing, China; ^3^School of Psychology, University of Queensland, BrisbaneQLD, Australia; ^4^Department of Psychology, James Cook University SingaporeSingapore, Singapore; ^5^Department of Psychology, Nanjing UniversityNanjing, China

**Keywords:** positive emotion, loving-kindness, compassion, Buddhism, meditation, four immeasurables

## Abstract

While it has been suggested that loving-kindness meditation (LKM) is an effective practice for promoting positive emotions, the empirical evidence in the literature remains unclear. Here, we provide a systematic review of 24 empirical studies (*N* = 1759) on LKM with self-reported positive emotions. The effect of LKM on positive emotions was estimated with meta-analysis, and the influence of variations across LKM interventions was further explored with subgroup analysis and meta-regression. The meta-analysis showed that (1) medium effect sizes for LKM interventions on daily positive emotions in both wait-list controlled RCTs and non-RCT studies; and (2) small to large effect sizes for the on-going practice of LKM on immediate positive emotions across different comparisons. Further analysis showed that (1) interventions focused on loving-kindness had medium effect size, but interventions focused on compassion showed small effect sizes; (2) the length of interventions and the time spent on meditation did not influence the effect sizes, but the studies without didactic components in interventions had small effect sizes. A few individual studies reported that the nature of positive emotions and individual differences also influenced the results. In sum, LKM practice and interventions are effective in enhancing positive emotions, but more studies are needed to identify the active components of the interventions, to compare different psychological operations, and to explore the applicability in clinical populations.

## Introduction

Loving-kindness meditation (LKM) is a special type of Buddhist meditation that aims to cultivate unconditional kind attitudes toward oneself and others. The core psychological operation is to keep generating one’s kind intentions toward certain targets, while the detailed operations vary across different Buddhist traditions. Generally, practitioners silently repeat some phrases, such as “may you be happy” or “may you be free from suffering” toward targets. In some traditions, they also visualize the mental image of the targets or light from one’s heart toward others to help the generation of intentions (Sujiva, 2007). The targets change gradually with practice, following an order from easy to difficult; they generally begin with oneself, then loved ones, neutral ones, difficult ones and finally all beings, with variations across traditions. Buddhism claims that LKM cultivates four sublime attitudes called “four immeasurables”: (1) loving-kindness, which refers to unselfish friendliness; (2) compassion, which refers to a willingness to cease the suffering of the distressed one; (3) appreciative joy, which refers to feeling happiness for other’s success or fortune; and (4) equanimity, which refers to calm toward the fate of others based on wisdom. It is worth noting that different sublime attitudes are cultivated by special subtypes of LKM, which are different in their psychological operations; for example, practitioners imagine suffering people to cultivate compassion in “compassion meditation,” and imagine happy people to cultivate loving-kindness in “LKM” in a narrow sense. To avoid the confusion of having two meanings of the term “LKM,” this article will use “LKM” in its broad sense, to refer to all of these subtypes of meditations, and will refer to certain subtypes of LKM as “LKM on loving-kindness” or “interventions that focus on compassion” and so on.

Empirical studies on LKM have increased sharply in the last 5 years (see Galante et al., 2014, for review), and one of the important outcomes of LKM is the enhancement of positive emotions. In particular, LKM was considered to be a way to provide continuous positive emotions and thereby outpace the “hedonic treadmill” effect (Diener et al., 2006) in which people return to their fixed emotional set-point after a temporary alteration of happiness (see Fredrickson et al., 2008). Previous narrative reviews on LKM that covered positive emotions concluded the following regarding LKM or its subtypes of meditations: they are “highly promising practices for improving positive affect” (Hofmann et al., 2011, p. 1131); it “can foster positive emotions” (Zeng et al., 2013, p. 1472); and it has “demonstrated significant improvements” (Shonin et al., 2015, p. 1) on positive emotions. A recent meta-analytic review that included LKM and other kindness-based meditations also concluded that LKM “facilitates positive emotions, although they are not entirely consistent” (Galante et al., 2014, p. 1109). In addition, other narrative reviews focused on the neural-mediator (Mascaro et al., 2015) and physiological effects (Shobitha and Kohli, 2015) of LKM, which will not be further discussed here.

It is worth noting that “emotion” and “affect” are often used interchangeably, as cited above, but these two concepts are different in the area of emotion study (see Fredrickson, 2001). The “emotion” can be conceptualized as “multicomponent response tendencies” (Fredrickson, 2001, p. 2) and “affect” can refer to consciously accessible feelings or subjective component of emotions. At the same time, “affect” can also refer to other affective phenomena like attitudes (Fredrickson, 2001). In the present review, the word “emotion” is limited to the subjective feeling of emotion, which is consistent with the concept in reviews mentioned above. According to the widely adopted “circumplex model of affect” which categorizes emotions with dimensions of valence (i.e., pleasant versus unpleasant) and arousal (Russell, 1980), the “positive emotions” in the present review refer to emotions with positive valence. As subjective feeling, positive emotions are measured with various self-report measurements. Some measurements assess the frequency of emotional experience in certain period of time with list of emotional words or short phrases (e.g., “proud,” “excited”; Watson et al., 1988), other measurements let responders rate some descriptions about positive emotions (e.g., “I consider myself a very happy person”; Lyubomirsky and Lepper, 1999).

The four previous reviews that included positive emotions (Hofmann et al., 2011; Zeng et al., 2013; Galante et al., 2014; Shonin et al., 2015) have some limitations. First, they did not focus on positive emotions and thereby did not comprehensively include studies with positive emotions. Hofmann et al.’s (2011) and Zeng et al.’s (2013) reviews were not based on systematic literature search, while Shonin et al. (2015) only included intervention studies with clinical samples or psychopathology-relevant outcomes. Galante et al. (2014) only selected randomized control trails (RCTs) and did not cover neuro-imaging studies. Second, their conclusions on positive emotions were based on narrative descriptions of individual studies, rather than a statistical summary across those mixed results. It is worth noting that the only statistical summary for effect on positive emotion in the meta-analysis by Galante et al. (2014) was combinations of two studies, which may have weaken the discussion. Third, the conclusion is general and many important variations among studies were not fully discussed. Shonin et al. (2015) noted that previous reviews mixed single-dose experimental design and long-term interventions, but the single-dose effect cannot be equivalent to the results of interventions. They also emphasized that the subtypes of LKM are different in terms of Buddhism, although they did not identify any difference between the subtypes of LKM in their review. Furthermore, recent studies have illustrated that LKM only enhances special types of positive emotions, such as other-focused (but not self-focused) positive emotions (Seppala et al., 2014).

The current literature on effect of LKM on positive emotions needs further clarification, this article attempted to provide some clarity by using a systematic review based on meta-analysis methodology. We would also separate out changes to the immediate positive emotions induced by the on-going practice of LKM and the daily positive emotions from the LKM interventions or long-term practice of LKM because they are different in nature. Changes in immediate positive emotions can be attributed to meditation practice here and now, whereas changes in daily positive emotions are usually the result of the influence of whole LKM interventions as well as other events in one’s life. Such a distinction is not the same as the distinction between the single-dose experimental design and the long term interventions proposed by Shonin et al. (2015); some studies involved long-term interventions (e.g., Klimecki et al., 2013, 2014) but evaluated the effect of the on-going practice of meditation in the laboratory, and thus, they are more similar to other studies that evaluated the immediate effect without interventions. In addition, we also examined by using subgroup analysis, meta-regression or sensitivity analysis the following important variables: (1) the psychological operations of loving-kindness and compassion; (2) the nature of positive emotions, such as other-focused versus self-focused positive emotions as mentioned above; (3) the structure and components of the interventions, including the entire length of interventions, the time spent on meditation practice, and the existence of weekly courses; and (4) clinical and non-clinical samples.

## Method

### Literature Search

The databases of Medline Plus (through June 5th, 2015), ISI Web of Knowledge (through June 5th, 2015), PsychInfo (1806 to June Week 1 2015, limited to peer-reviewed journal), Embase (through June 4th, 2015), CINAHL Plus (through June 5th, 2015), AMED (through May 2015) and Cochrane Central Register of Controlled Trials (through May 2015) were used. The keywords for the search were “(loving AND kindness) OR compassion OR ((Appreciating OR Appreciative OR Sympathetic OR Empathic) AND Joy) OR equanimity OR metta OR mudita OR karuna OR upekkha” combined with “Meditat^∗^ OR Buddhis^∗^”, which were adjusted for different databases.

### Selection of Studies and Outcomes

The inclusion criteria were (a) articles published in a peer-reviewed journal in the English language; (b) empirical studies that focused on LKM; (c) studies with any self-report measurements on positive emotions as outcome variables; and (d) studies with either clinical or non-clinical samples. The exclusion criteria were: (a) articles not published in peer-reviewed journal in the English language; (b) studies without empirical data; (c) not LKM, or interventions where LKM accounted for less than 50% of major practices; (d) did not use outcome positive emotion measurements. It is worth noting that (1) the practices that induce feelings of kindness through the imagination of sacred god (e.g., Engström and Söderfeldt, 2010) or receiving love from others (e.g., compassion focused imaging; Judge et al., 2012) were not considered as LKM because LKM was characterized by sending kind intentions toward targets as mentioned above; (2) the self-report measurements that reflected more attitudes and behaviors than subjective feelings (e.g., savoring; Johnson et al., 2011; compassionate love; Leiberg et al., 2011) were excluded although some authors considered them as positive affects or positive experiences.

### Data Extraction and Synthesis

Two authors (XZ, CC) independently reviewed the titles and abstracts to exclude duplications and irrelevant studies; the full text was obtained if the study was considered to be relevant or not clear by either of the two authors. The same two authors independently reviewed the full text to identify whether the study was an empirical study focused on LKM and then extracted measurements of positive emotions used. Discrepancies were discussed until a consensus was reached with consultation from the third author (RW). The reference list of identified empirical studies and previous reviews on LKM were checked for missing studies.

### Strategy for the Meta-analysis

The meta-analysis was conducted by Comprehensive Meta-Analysis 3. All of the studies in the present meta-analysis reported outcomes with continuous variables, and the effect sizes were transformed into standardized mean differences (Hedges’ *g*) with 95% CI (Hedges and Olkin, 1985), which can be interpreted as 0.2 for small, 0.5 for medium, and 0.8 for large effect sizes (Cohen, 1988). The effect size and its variance were calculated in the ways that they could be most precisely evaluated based on the available data for each study (see Supplementary Material for details). For studies with multiple time points, only post-intervention data rather than longitudinal data were used because the longitudinal data were few and the time intervals were varied across studies. For the studies with multiple measurements of positive emotions, the meta-analysis reports the range of estimation rather than combining different measurements because some measurements are not suitable for combination.

Meta-analyses were conducted with at least two studies of same comparison, and random effect models were used because these studies were varied in several ways. For meta-analyses that included more than five studies, a statistical evaluation of heterogeneity was conducted based on the Q value and I2 statistics, potential publication bias was evaluated with the classic Fail-safe N and funnel plot, and sensitivity analysis was conducted with one study excluded each time. Furthermore, subgroup analyses for psychological operations and existence of weekly courses were conducted with at least two studies in each subgroup, and the within-subgroup variance was pooled across subgroups to ensure precise evaluation. Meta-regressions for the length of interventions were conducted with at least five studies. Sensitivity analyses were conducted with all studies with clinical samples excluded.

## Results

### Search Results and Characteristic of Studies

**FIGURE 1:**
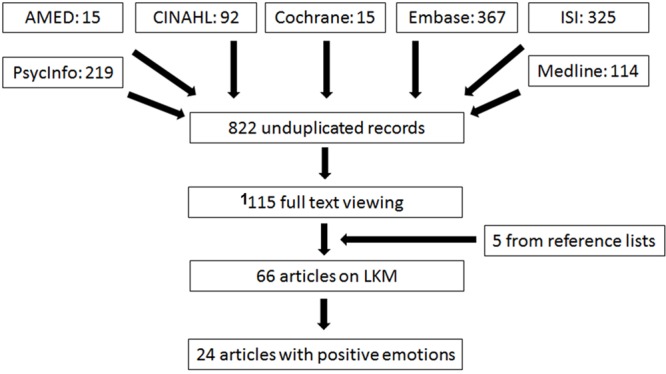
**Flowchart for the literature search**.

The flowchart of the literature search is shown in **Figure 1**. Sixty-six articles were identified as empirical studies on LKM from 115 records for full text viewing (see Supplementary Material for reasons for exclusion). Among them, 24 articles included self-reported positive emotions and were reviewed below. Of note, 10 out of these 24 articles were not included in any two previous systematic reviews (Galante et al., 2014; Shonin et al., 2015).

The article of Cohn and Fredrickson (2010) reported the longitudinal data of Fredrickson et al. (2008), while two articles (Alba, 2013; Neff and Germer, 2013) reported two independent interventions; therefore, there are 25 independent studies in total. Among them, 10 RCT studies (**Table 1**) and 7 non-RCT studies (**Table 2**) evaluated the effect of the interventions on daily positive emotions, 6 RCT studies evaluated the immediate effect of on-going practice of LKM (**Table 3**). The remaining two studies (**Table 4**) included one within a subject study that evaluated the on-going practice of LKM (Hutcherson et al., 2015) and one cross-sectional study that compared LKM experts with novices (Lee et al., 2012). These are not included in the meta-analysis section below. The risk of biases for each study can be found in Supplementary Material; these included 38.9% high risk, 25.7% low risk and 35.4% unclear risk.

**Table 1:** Randomized control trail (RCT) studies that evaluated the effect of intervention on daily positive emotions.

Study	LKM	Control	Subjects	Measurements
Fredrickson et al., 2008; Cohn and Fredrickson, 2010	One hour weekly course for 6 week, focused on loving-kindness.	Wait list	Healthy adults from company, 202 ITT to 139 completer (65.5% female, 41 ± 9.6 years old; 67 in LKM group), 95 presented in 15 months follow up measurement. Four has previous meditation experience.	Modified Differential Emotions Scale (Daily report) (A)
Jazaieri et al., 2014	Two hours course for 8 weeks, focused on compassion.	Wait list	Healthy community adults, 100 ITT (60 in LKM group with 39 females, 41.98 ± 11.48 years old; 40 in wait list group with 33 females, 44.68 ± 13.05 years old) to 80 completers (50 in LKM group), no follow up measurement. Unknown previous meditation experience.	Subjective Happiness Scale
Kok et al., 2013	Two hours course for 8 weeks, focused on compassion.	Wait list	University staff, 71 intent to treat and 65 completers (66% female with 37.5 years of median age; 31 in LKM group), no follow up measurement. No previous meditation experience.	Modified Differential Emotions Scale (Daily report)
Koopmann-Holm et al., 2013	Two hours course for 8 weeks, focused on compassion.	(1) Matched mindfulness meditation (MM); (2) Matched theater therapy (TT); (3) No intervention (NI)	Healthy adults, 96 ITT to 74 completers (all females; 21.13 ± 3.49; 17 in LKM group; 19 in MM group; 16 in TT group; 22 in NI), no follow up measurement. No previous meditation experience.	Affect Valuation Index
Leiberg et al., 2011	One day (6 h) training, focused on compassion.	Matched memory training	Healthy adults, 69 ITT (all females; 25.18 ± 4.08 years) to 59 completers (27 in LKM group), no follow up measurement. No previous meditation experience.	Positive and Negative Affect Scale (unknown time)
May et al., 2014	Five weeks self-training without course, focused on loving-kindness.	Matched concentration meditation	University students, 31 ITT (71% females, age unknown) to *N* = 29 (15 in LKM group). Multiple ABA design, ceasing meditation for 3 weeks. Unknown previous meditation experience.	Positive and Negative Affect Scale (past few days)
Neff and Germer, 2013; Study 2	Two hours per week for 8 week, plus 4 h boost session, focused on compassion.	Wait list	Healthy community adults, 54 intent to treat to 51 completers, (24 in LKM group, 78% female, 51.21 ± 12.02; 82 females, 49.11 ± 11.59 in wait list group), all 24 in LKM group presented in 3 month follow up measurement. 78% had previous meditation experience.	Subjective Happiness Scale
Schutte, 2014	Three weeks self-training with weekly scripts, focused on loving-kindness.	Wait list	Healthy adults, 408 ITT (54.7% females, 38.31 ± 15.10) to 374 completers (200 in LKM group), no follow up measurement. Unknown previous meditation experience.	Positive and Negative Affect Scale (past month)
Shahar et al., 2014	Ninety minutes course for 7 weeks, focused on loving-kindness.	Wait list	Adults with high self-criticism, 19 ITT (14 females with 28.68 ± 10.37 yeas) to 14 completer in LKM; 19 ITT (nine females with 32.56 ± 10.68 yeas) to 17 completer in wait list group, 20 presented in 3 months follow up measurement. No previous meditation experience.	Positive And Negative Affect Scale (last week)
Weytens et al., 2014	Two hours course for 6 weeks (post-measurement at 4th week), focused on loving-kindness.	(1) Positive Emotion Regulation (PER); (2) Wait list (WL)	Healthy university students, 113 ITI (77.9% females, 22.29 ± 2.49 years old) to 79 completer (16 in LKM group, 28 in PER, 35 in WL), no follow up measurement. Unknown previous meditation experience.	Subjective Happiness Scale

*(A) This study also used the day reconstruction method to measure positive emotions. But this part was not included in the meta-analysis because it was only measured in post-measurement.*

**Table 2:** Non-RCT studies that evaluated the effect of intervention on daily positive emotions.

Study	LKM	Subjects	Measurements
Alba, 2013; Study 1	Four days retreat, focused on loving-kindness.	Community sample, 23 ITT to 20 completers, gender and age unknown, 13 presented in 2 weeks follow up measurement. Unknown previous meditation experience.	Fordyce Emotions Questionnaire
Alba, 2013; Study 2	Ten days retreat, focused on loving-kindness.	Community sample, 39 ITT (28 females with 50.21 ± 14.41 years old) to 31 completers, 15 presented in 2 weeks follow up measurement. Unknown previous meditation experience.	Fordyce Emotions Questionnaire
Johnson et al., 2011	One hour weekly course for 6 weeks, with boost session after 6 week, focused on loving-kindness.	People with schizophrenia. 18 ITT (17% females, 29.4 ± 10.2 years old) to 16 completers, 14 presented in 3 months follow up. Unknown previous meditation experience.	Modified Differential Emotions Scale (Past 2 weeks) Day Reconstruction Method (certain day)
Kearney et al., 2014	Ninety minutes course for 12 weeks, focused on loving-kindness.	People with PTSD. 42 ITT (41.9% females, 53.6 mean ages) to 37 completers, 34 presented in 3 month follow up. More than half have various meditation experiences.	Circumplex Measure of Emotion (past 7 days)
May et al., 2011	Self-training for 8 weeks without course, focused on loving-kindness.	University students, *N* = 13 (76.9% females mean age 22.08), no follow up measurement. Unknown previous meditation experience.	Positive And Negative Affect Scale (unknown time)
Neff and Germer, 2013; Study 1	Two hours per week for 8 week, plus 4 h boost session, focused on compassion.	Health community adults, 23 ITT to 21 completers (95% females, 51.26 ± 11.28 years old), no follow up measurement (for positive emotion). Eighty one percent have previous meditations experience.	Subjective Happiness Scale
Sears and Kraus, 2009	No course, 15 min per week for 12 weeks, covered four immeasurables.	University students, 20 ITT to 17 completers (59% females with 22.80 ± 6.86 in whole sample of study), no follow up measurement. Unknown previous meditation experience.	Positive And Negative Affect Scale (past week)

**Table 3:** RCT studies that evaluated the effect of on-going practice of LKM.

Study	LKM	Control	Subjects	Measurements
Hutcherson et al., 2008	Received love from others and sent loving-kindness to others, 8 min practice.	Neutral visualization	Healthy adults, *N* = 93 (53 female, mean age 23.6 years, 45 in LKM group), no or very little previous meditation experience.	Positive affects (combined calm, happy, loving)
Seppala et al., 2014	Received love from others and sent loving-kindness to others, 8 min practice.	(1) Neutral visualization (2) Induction of Pride	Healthy adults, *N* = 134 (59% female, mean age 19 years, 46 in LKM, 44 in each control group), unknown previous meditation experience.	Self-focused positive emotions (proud, self-esteem, self-satisfaction) and other-focused positive emotions (friendly, close to others, affection, loving)
Klimecki et al., 2013	Sent compassion to people in video (Previous 8 h of LKM training).	Memory skill (Participants received 8 or more hours of training)	Health adults, *N* = 58 (all females, 24.3 ± 4.7 years in sample of whole research; 28 in LKM group), no previous meditation experience.	Positive affect when seeing suffering in video (single item)
Klimecki et al., 2014	Sent compassion to people in video (Previous 8 h of empathy training and then 8 h of LKM training).	Memory skill (Participants received two 8 or more hours training on memory skill.)	Health adults, *N* = 53 (all females, 25 with 25.88 ± 4.32 years in LKM group; 28 with 22.89 ± 4.02 years in memory group), no previous meditation experience.	Positive affect when seeing suffering in video (single item)
Wheeler and Lenick, 2014	Sent compassion to oneself and others, 25 min practice.	Music	Health adults, *N* = 62 (71% female, 19.7 mean age, 32 in LKM group), no previous meditation experience.	Positive And Negative Affect Scale (current moment)
Feldman et al., 2010	Sent compassion to oneself and others, 15 min practice.	(1) Mindfulness breath (MB), (2) Progressive muscle relaxation (PMR)	Health adults, *N* = 190 (all females, 19.83 ± 1.34 years old; 59 in LKM, 68 in MB, 63 in PMR), 78.7% no previous meditation experience.	Positive and Negative Affect Scale (“right now”)

**Table 4:** Studies with other designs.

Study	Design	Conditions	Subjects	Measurements
Hutcherson et al., 2015	Within subject design, effect of on-going practice	LKM: 3^∗^ 2.25-min guided LKM (Received love from others and sent loving-kindness to others), no previous intervention. Control: matched neutral visualization.	University students, *N* = 19 (11 females, 20.9 ± 4.1 years old), 5 with little experience on concentration meditation.	Social connection positive emotions (friendly, loving, happy, joyful); Self-focused positive emotions (self-esteem, being proud)
Lee et al., 2012	Cross-sectional comparison, effect on daily emotions	Comparison between LKM meditators and matched novices.	LKM meditators: *N* = 11 (all males, 51.82 ± 11.28 years old), with 7,491.98 ± 6,681.43 h experience of LKM. Novices: *N* = 11 (all males, 47.34 ± 8.95 years old), with no experience of LKM.	Chinese Affect Scale

### Meta-analyses

#### RCT Studies that Evaluate Daily Positive Emotions

**FIGURE 2:**
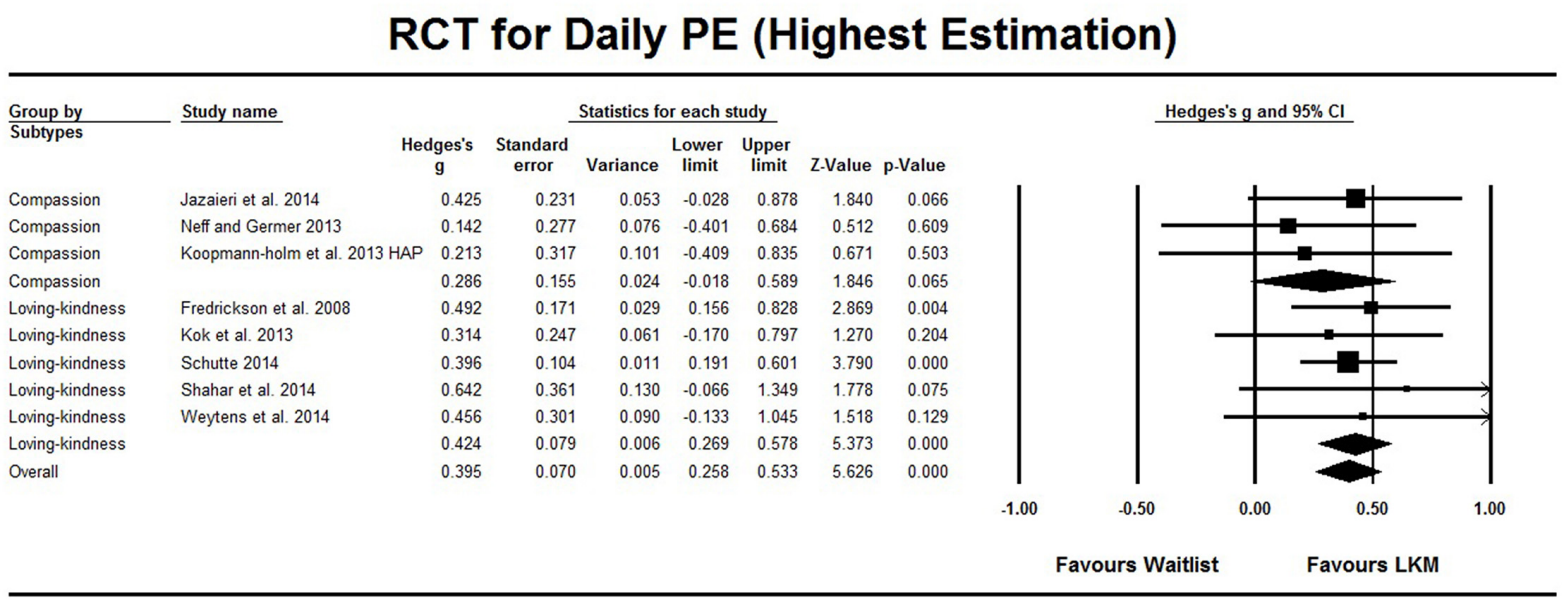
**Randomized control trail (RCT) studies on daily PE, comparison with waitlist control group**. The highest estimation was based on high arousal positive (HAP) emotion in Koopmann-Holm et al. (2013). Subgroup analysis compared LKM on loving-kindness and LKM on compassion.

**FIGURE 3:**
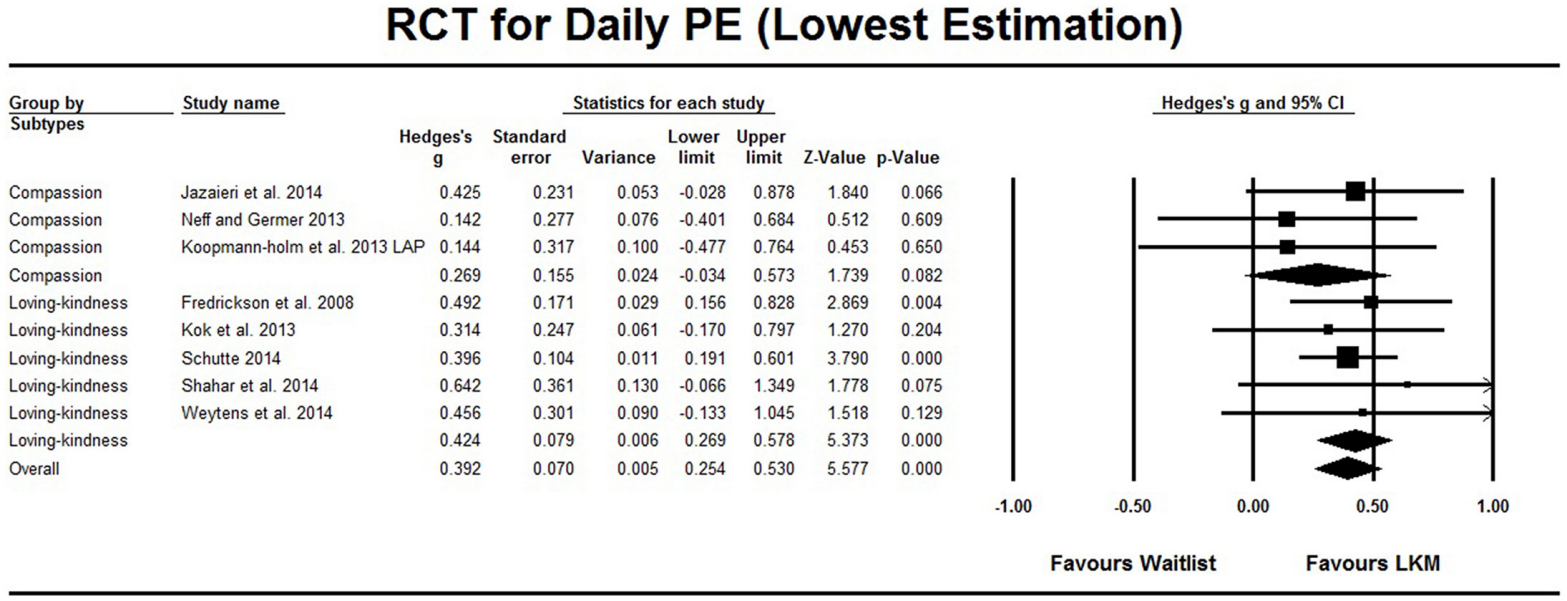
**RCT studies on daily PE, comparison with waitlist control group.** The lowest estimation was based on low arousal positive (LAP) emotion in Koopmann-Holm et al. (2013). Subgroup analysis compared LKM on loving-kindness and LKM on compassion.

As shown in **Table 1**, among the 10 RCT studies that evaluated the effect of the LKM intervention on daily positive emotions, eight studies involved a comparison between LKM and the wait list control group. Koopmann-Holm et al. (2013) distinguished between high arousal positive (HAP) emotions and low arousal positive (LAP) emotions, and the Hedges’ *g* were 0.395 (95%CI [0.258, 0.533], **Figure 2**) and 0.392 (95%CI [0.254, 0.530], **Figure 3**), respectively. The heterogeneity test showed a *Q* value of less than *df*, which indicated that no significant heterogeneity was found. An evaluation for publication bias showed a classic *Fail-safe N* is larger than 44, which referred to a low risk of publication bias (the funnel plot can be found in Supplementary Material). Sensitivity analysis showed the effect sizes did not essentially change when one study was excluded from meta-analysis (see Supplementary Material for details).

Furthermore, subgroup analysis on the difference between interventions that focused on loving-kindness and those that focused on compassion (see **Table 2**, eight studies) showed no significant difference (*p* > 0.374), but the interventions that focused on loving-kindness had a medium effect (Hedges’ *g* = 0.424, 95% CI [0.269, 0.578]), while the effect sizes for interventions focused on compassion had lower estimated points, and its 95% CI included the 0 point (Hedges’ *g* = 0.286, 95% CI [-0.018, 0.589] or 0.269, 95% CI [-0.034, 0.573]), see **Figures 2** and **3**. Meta-regressions across the eight comparisons were conducted to explore the relationships between the effect and the length of interventions, and the results showed that neither the total length of the intervention (e.g., 6 weeks; available in eight studies) nor the time of intervention (e.g., 6 weeks with 2 h per week; available for seven studies) could predict effect size (*p* > 0.349). The subgroup analysis for the existence of weekly courses (existed versus not existed) was not conducted, because all of these interventions consisted of weekly courses or self-guide scripts. In addition, Shahar et al. (2014) evaluated LKM interventions among a sub-clinical sample with high self-criticism and reported a significant result; the effect size based on other studies with non-clinical samples did not essentially change as mentioned above.

In addition to the comparisons with the wait list control group, three studies did not find significant differences between LKM and four different active control groups, including mindfulness meditation (Koopmann-Holm et al., 2013), theater therapy (Koopmann-Holm et al., 2013), memory training (Leiberg et al., 2011), and positive emotion regulation (Weytens et al., 2014). Only May et al. (2014) reported that LKM showed a significantly larger increase in positive emotions than concentration meditations (see Supplementary Material for the effect sizes of these comparisons).

#### Non-RCT Studies that Evaluate the Daily Positive Emotions

**FIGURE 4:**
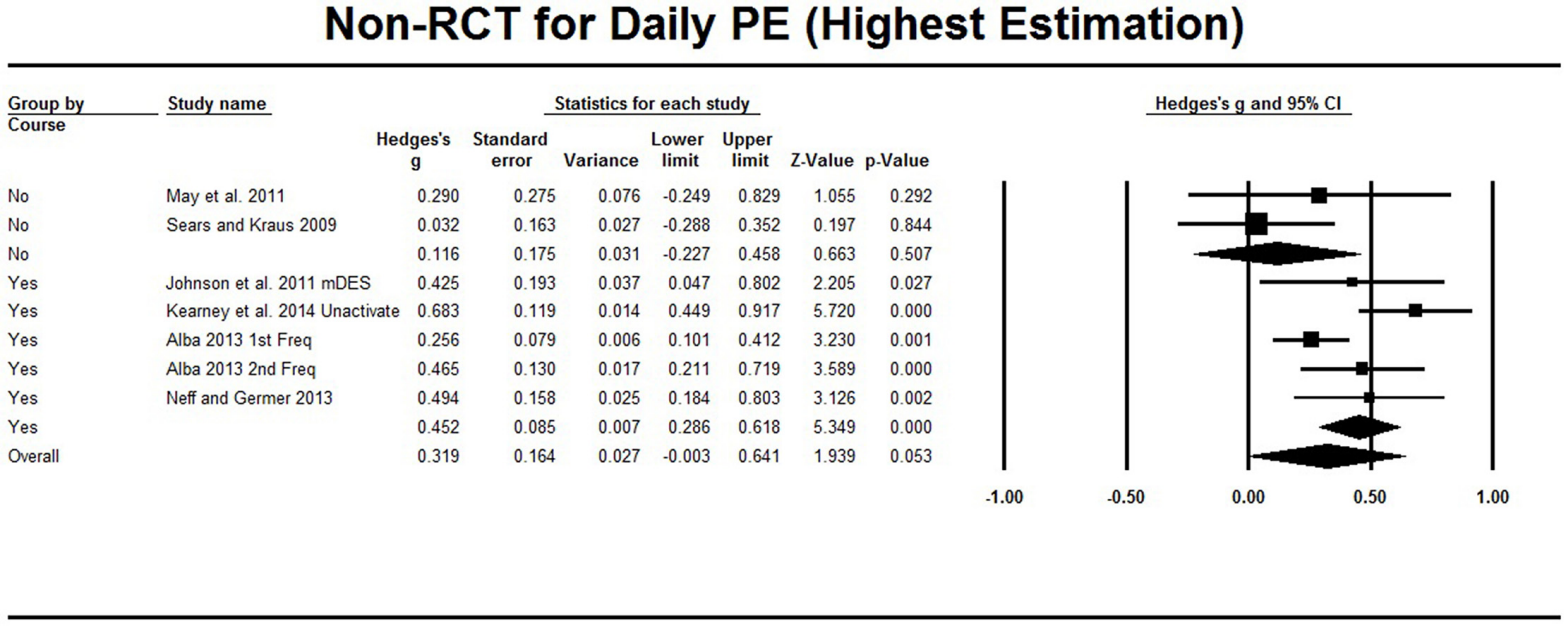
**Non-RCT studies on daily PE.** The highest estimation was based on frequency of happiness (Freq) in Alba (2013), unactivated positive emotion (Unactivate) in Kearney et al. (2014) and modified Differential Emotions Scale (mDES) in Johnson et al. (2011). Subgroup analysis compared LKM with course and without course.

**FIGURE 5:**
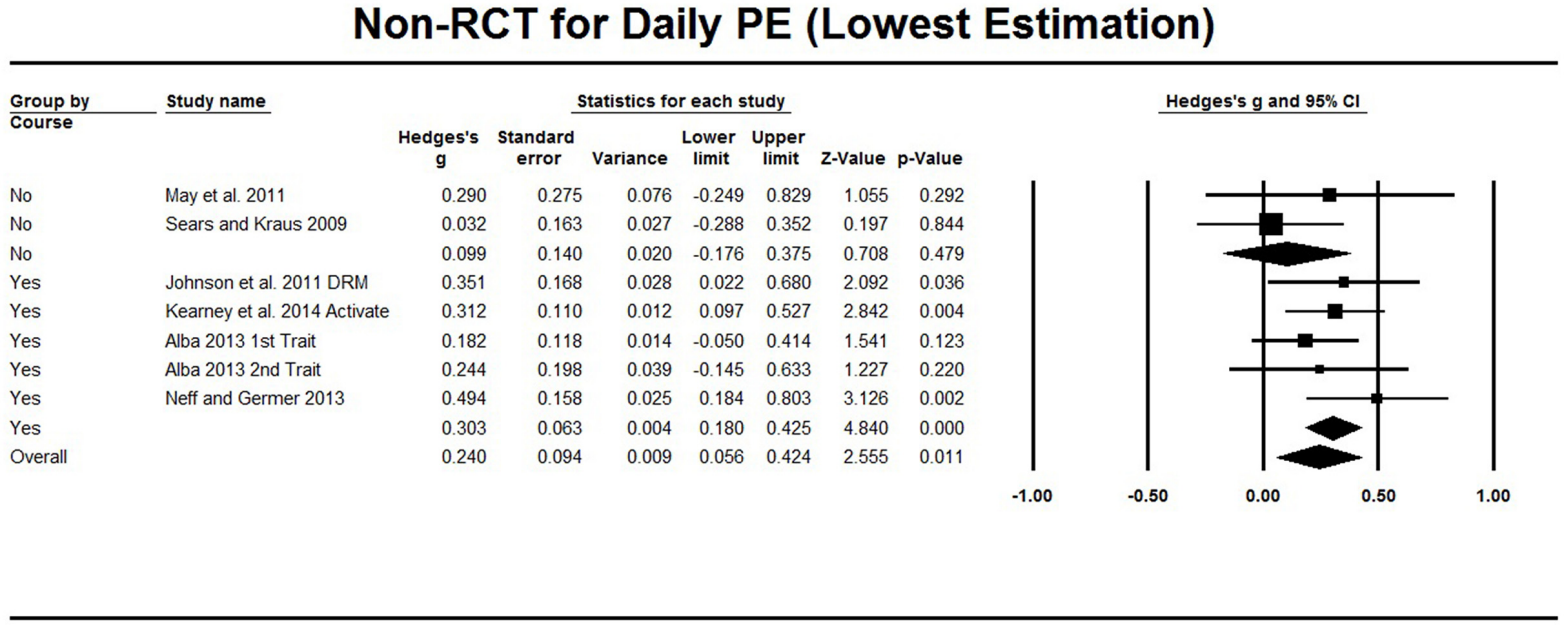
**Non-RCT studies on daily PE.** The lowest estimation was based on long-term happiness (Trait) in Alba (2013), activated positive emotion (Activate) in Kearney et al. (2014), day reconstruction method (DRM) in Johnson et al. (2011). Subgroup analysis compared LKM with course and without course.

For seven non-RCT intervention studies, meta-analysis was conducted based on the single group pre–post comparison. As shown in **Table 2**, Kearney et al. (2014) distinguished activated and unactivated positive emotions, Alba (2013) measured the frequency of happiness and trait-like long-term happiness, and Johnson et al. (2011) measured both the frequency and intensity of positive emotions. The meta-analysis showed that the highest Hedges’ *g* = 0.319 (95% CI [-0.003, 0.641], **Figure 4**) and that the lowest Hedges’ *g* = 0.287 (95% CI [0.134, 0.440], **Figure 5**). The heterogeneity test also showed a *Q* value of less than *df*, and therefore, no significant heterogeneity was found. Classic *Fail-safe N* was larger than 31, which conferred a low risk of publication bias (the funnel plot can be found in Supplementary Material). Sensitivity analysis showed the effect sizes did not essentially change when one study was excluded from meta-analysis (see Supplementary Material for details).

Furthermore, subgroup analysis for psychological operations was not conducted because only Neff and Germer (2013) used the intervention focused on compassion while other studies used interventions focused on loving-kindness. Neff and Germer (2013) reported significant increases in positive emotion, and exclusion of this study did not essentially change the effect size as mentioned above. The influence of the length of the interventions was not explored because the structure of interventions varied from intensive whole day meditation for 4 days (Alba, 2013) to 15 min per week but lasting 12 weeks (Sears and Kraus, 2009). As for the influence of weekly courses, subgroup analysis showed that two studies (Sears and Kraus, 2009; May et al., 2011) that did not include weekly courses showed very low effect size (Hedges’ *g* = 0.116, 95% CI [-0.249, 0.829]), whereas the effect size for the other five studies was relatively higher (Hedges’ *g* = 0.452, 95% CI [0.286, 0.618]) or 0.303, 95% CI [0.180, 0.425]), although the between subgroup difference was not significant (*p* > 0.083) (see **Figures 4** and **5**). In addition, Johnson et al. (2011) and Kearney et al. (2014) applied the interventions among people with schizophrenia and people with PTSD, respectively; both of them reported significant increases in positive emotions, and the effect size based on other studies with non-clinical samples did not essentially change (Hedges’ *g* = 0.264, 95% CI [0.023, 0.504] or 0.229, 95% CI [0.060, 0.398]).

#### RCT Studies that Evaluate the Effect of On-going Practice of LKM

**FIGURE 6:**
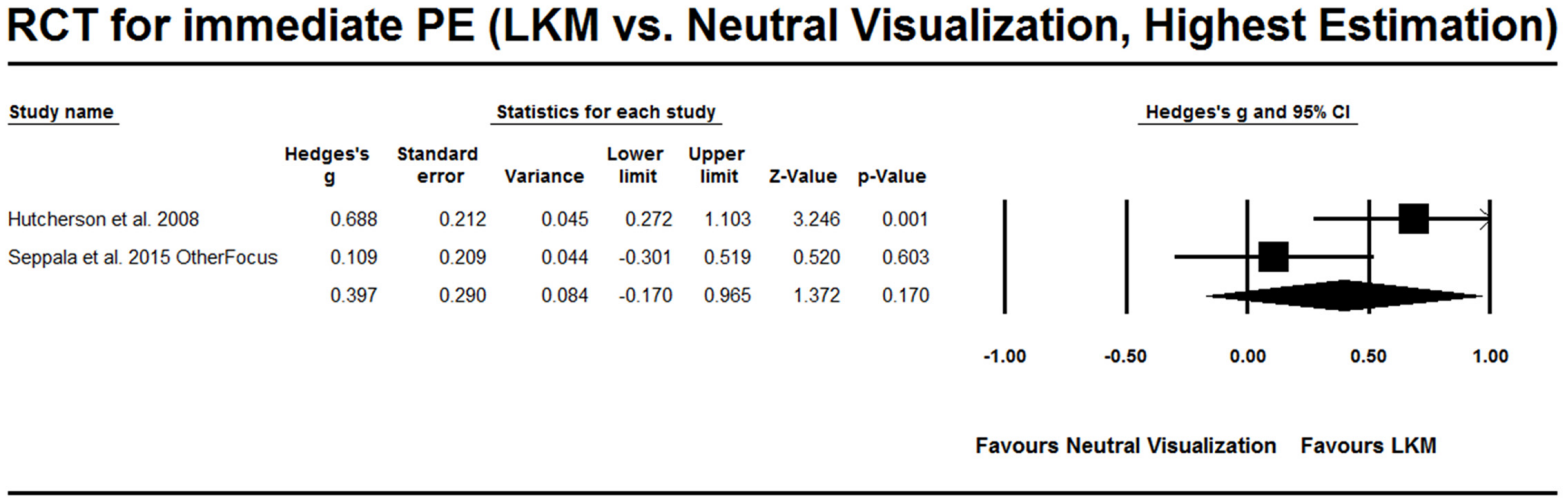
**RCT studies for PE from on-going practice, comparison with neutral visualization.** The highest estimation was based on prosocial positive emotion (OtherFocus) in Seppala et al. (2014).

**FIGURE 7:**
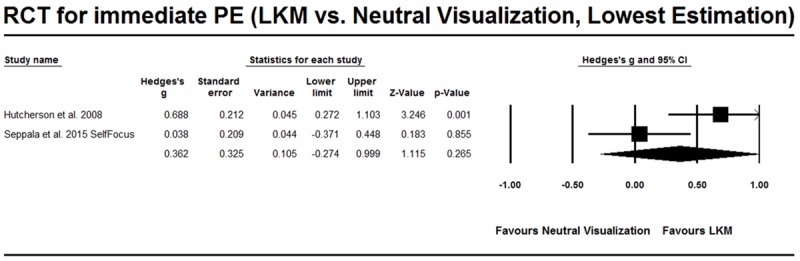
**RCT studies for PE from on-going practice, comparison with neutral visualization.** The lowest estimation was based on self-focused positive emotion (SelfFocus) in Seppala et al. (2014).

As shown in **Table 3**, six RCT studies evaluated the effect of the on-going practice of LKM and their comparisons or tasks varied. The studies by both Hutcherson et al. (2008) and Seppala et al. (2014) involved a comparison between LKM and neutral visualization, and Seppala et al. (2014) further discriminated between other-focused positive emotions and self-focused positive emotions. The result of the meta-analysis showed that Hedges’ *g* = 0.397, 95%CI [-0.170, 0.965] (**Figure 6**), when other-focused positive emotions were selected, and Hedges’ *g* = 0.362, 95%CI [-0.274, 0.999] (**Figure 7**), when self-focused positive emotions were selected in Seppala et al. (2014).

**FIGURE 8:**
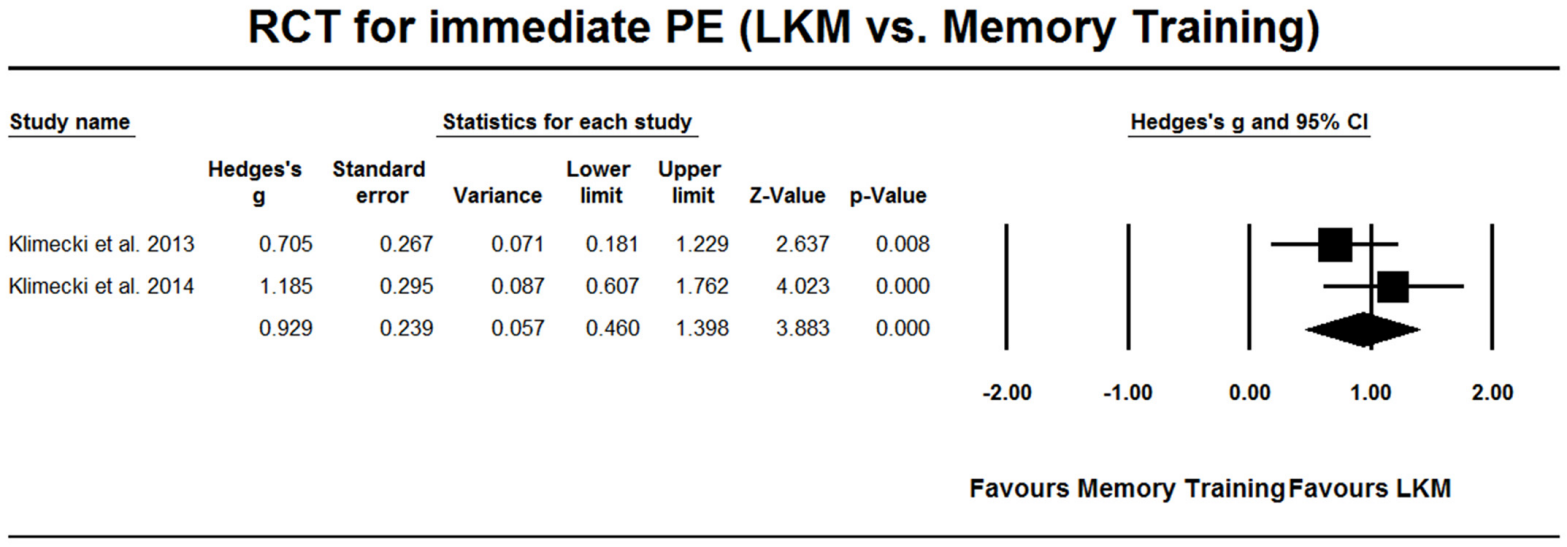
**RCT studies for PE from on-going practice, comparison with memory training**.

Klimecki et al. (2013, 2014) compared LKM intervention with memory training and evaluated how people, when actively applying their skill, reacted to videos of individuals who were suffering. The result of the meta-analysis showed that LKM had a large effect size in comparison with memory training (Hedges’ *g* = 0.929, 95%CI [0.460, 1.398], **Figure 8**).

Other comparisons only consisted of one study each, as shown in **Table 1**. Seppala et al. (2014) reported that the LKM condition showed significantly greater other-focused positive emotions than the pride induction condition (Hedges’ *g* = 0.200, 95%CI [-0.211, 0.611]), whereas the pride condition showed significantly greater self-focused positive emotions than the LKM group (Hedges’ *g* = –0.755, 95%CI [-1.179, -0.330]). Wheeler and Lenick (2014) reported that the LKM condition did not differ significantly from the music control condition (Hedges’ *g* = 0.014, 95%CI [-0.478, 0.506]). Similarly, Feldman et al. (2010) reported that the LKM condition showed significantly greater positive emotions in comparison with mindfulness meditation but not significantly greater positive emotions in comparison with progressive relaxation (effect size not available).

## Discussion

### General Results of the Reviewed Studies

#### Effect of LKM Interventions on Daily Positive Emotions

The majority of the reviewed studies (17 out of 25) investigated the effect of LKM interventions on daily positive emotions. Both RCT studies with a wait list control group and non-RCT studies showed that LKM interventions had medium effect sizes, although the results are inconsistent across all studies. There are only four comparisons between LKM and active control groups, and only May et al. (2014) reported that LKM showed a greater improvement than concentration meditation. That is, whether LKM interventions are better than active interventions is still not clear and requires more studies. In addition, all of the studies that measured follow-up results (see **Tables 1** and **2**) reported that positive emotions gained at post intervention were maintained.

It is cautioned here that at least two factors may over-estimate the results of the interventions above. The first factor is the expectancy effect. Non-RCT studies and RCT studies with a wait list control group can hardly control this effect. In particular, LKM explicitly guides practitioners to seek a feeling of warmth or imagine the smile of the target. As well the philosophy that gaining happiness from good interpersonal relationships is also an important idea of LKM (Sujiva, 2007). Empirically, Koopmann-Holm et al. (2013) reported that LKM (and also mindfulness meditation) enhances the value of, but not the actual experience of LAP emotion, and they argued that findings on emotional change due to meditation may come from the expectancy effect. In addition, Cohn and Fredrickson (2010) mentioned that participants in the wait list group had less practice at home when they received an intervention, which also indicates the influence of experimental manipulation on participants’ motivation. The second factor is the problem of self-selection. People who are interested in meditation are more willing to accept the interventions. Consistently, some studies have a high proportion of participants with previous meditation experience (e.g., Neff and Germer, 2013), and Weytens et al. (2014) reported a high dropout rate, with many participants finding themselves unsuitable for meditation. Another problem associated with self-selection is a lack of intent-to-treatment (ITT) analysis. The completers may be more suitable for intervention or have more motivation than participants who drop out. In addition to the common factors above, other problems that summarized the risk of biases evaluation may also have led to over-estimation in certain studies (see Supplementary Material). For example, the LKM group had higher baseline positive emotions than the concentration meditation group in the study of May et al. (2014), and other studies reported that baseline positive emotions influenced the effect of LKM intervention (Kok et al., 2013).

In summary, it is reasonable to conclude that LKM interventions can increase self-reported positive emotions of some participants, which is consistent with the conclusions of previous reviews (Hofmann et al., 2011; Zeng et al., 2013; Galante et al., 2014; Shonin et al., 2015). However, this effect cannot be generalized to other people with different characteristics, and the portion of expectation or demanding effect is unknown.

#### Effect of the On-going Practice of LKM

Six RCT studies and one within subject study evaluated the immediate effect of the on-going practice of LKM. The tasks, control conditions and introduction of LKM varied across studies, and the results also varied from no significant difference to large effect sizes. These studies suffered lesser methodological problems in comparison with studies on interventions (see risk of biases evaluation in Supplementary Material). Particularly, most studies did not involve previous interventions and covered the purpose of study to the participants, and relatively simple tasks in laboratory settings also excluded many confounding factors. Therefore, the existence of greater positive emotions in comparison with emotionally neutral conditions (e.g., neutral visualization; memory training) supported the idea that the successful practice of LKM can induce positive emotions. In contrast, the reasons why LKM did not show better results in some studies could be complex. One possibility that is worth noting is that the introduction of LKM did not correctly guide practitioners who practiced LKM for the first time, as noted by Crane et al. (2010).

Furthermore, because participants practiced LKM for the first time or only received short-term training (e.g., Klimecki et al., 2013), the present estimation of the effect should be limited to novices, and whether long term practice improves the effect has not yet been confirmed. Klimecki et al. (2013) found that practice time could not predict effect in their studies, but they noted the self-reported practice time at home lacked reliability. Of note, Lee et al. (2012) and the works of several other research teams (e.g., Garrison et al., 2014) confirmed the difference in brain activation during meditation between meditators and novices, but none of them recorded the subjective feelings of emotion during meditation and therefore could not directly answer whether long-term practice improves the effect of on-going practice.

### Potential Factors that Influence the Results

The discussion above focused on the overall effect of LKM, but the studies, especially their interventions, varied in many aspects. The subgroup analysis and meta-regression did not find any significant results, but it is worth noting that the powers of these analyses are generally low. Nevertheless, the meta-analysis still indicated that some factors may influence the effects, and some individual studies also provided information on these issues, which will be discussed below.

#### Difference in Psychological Operations

The meta-analysis for interventions with wait list control groups found that interventions that focused on loving-kindness showed a medium effect size, but interventions that focused on compassion showed a relatively lower estimated effect size. It addition, interventions that focused on compassion did not show better results than the active control groups (Leiberg et al., 2011; Koopmann-Holm et al., 2013), whereas the results for interventions that focused on loving-kindness were mixed (May et al., 2014; Weytens et al., 2014). Although the number of studies was small and the studies varied in many aspects, such results nevertheless implied a potential difference between these two sub-types of LKM.

At least two reasons can explain why interventions on loving-kindness tend to enhance more positive emotion than interventions on compassion. First, the emotional experience during meditation is different. Practitioners often imagine others smiling when cultivating loving-kindness while they imagine suffering people to cultivate compassion; some previous articles also reported that some practitioners even begin to cry when cultivating compassion (Lutz et al., 2009). Second, the positive emotions were less emphasized in interventions on compassion. Many interventions on loving-kindness emphasize the idea of achieving personal happiness through feelings of love or good relationships with others, whereas interventions on compassion often encourage one to pay attention to and take care of suffering people, even in the midst of one’s own suffering (Lutz et al., 2009). The discussion above has mentioned the influence of the expectancy effect; therefore, different emphases between interventions may also influence the results.

In addition, all of the reviewed studies involved LKM for both self and others to some extent and did not compare differences between targets. Therefore, no evaluation for this aspect of difference can be conducted. However, three studies in the present review replaced blessing for oneself with receiving love from others (Hutcherson et al., 2008, 2015; Seppala et al., 2014), which are different from LKM. In particular, Zeng et al. (2013) noted that achieving happiness through self-kindness versus outside kindness is significantly different in terms of Buddhist philosophy. The present review included these three studies considering that they involved the practice of LKM (toward others) and were widely accepted as LKM in previous studies, but future studies should investigate whether such differences in psychological operations influences the effect on positive emotions or other outcomes.

#### Variation in the Length of Intervention, Meditation Practice, and Weekly Courses

The present meta-analysis explored the relationship between the length of interventions and the effect on daily positive emotions, but no significant relationship was detected. This can be partially attributed to the low power of analysis and limited number of studies, but it is also possible that such indicators at the study level were not sensitive predictors. After all, the structure of interventions varies in many aspects, and many details were not reported by every study; therefore, it is hard to compare studies across study levels.

The present meta-analysis did not explore the relationship between the required or actual time spent on meditation practice because many studies did not report this information. However, some studies reported the correlation between the time of meditation and effect at the individual level. Three studies reported that there was no significant relationship between practice time and daily positive emotions in their interventions (Leiberg et al., 2011; May et al., 2011; Jazaieri et al., 2014). In addition, Lee et al. (2012) reported that LKM meditators did not have greater positive emotions in daily life than matched novices, and there was no significant relationship between practice time and daily positive emotions among meditators. Of note, the null results may be due to the low reliability of self-reported practice time, as noted by Klimecki et al. (2013), but it is also worth noting that some studies found significant relationships between practice time and other results (Leiberg et al., 2011). It is also possible that the quality of practice is more important than the length of practice because several studies mentioned individual differences regarding reactivity toward LKM (Cohn and Fredrickson, 2010; Kok et al., 2013), which implied that people gain different improvements with equal practice time. From another perspective, Fredrickson et al. (2008) and Kok et al. (2013) recorded meditation practice and positive emotions every day and reported that the positive emotions gained from each hour of practice increased with intervention. Fredrickson et al. (2008) also illustrated that LKM on certain mornings can enhance the gain of positive emotions from social interactions on the same morning after intervention. Based on longitudinal results, Cohn and Fredrickson (2010) further reported that whether participants maintained LKM practice did not influence long-term positive emotions, and explained that positive emotions in the long term might be maintained by other improvements in daily life (e.g., better interpersonal relationships). In summary, existing studies confirmed that LKM can provide immediate positive emotions when practiced and also boost daily positive emotions, at least in the short term, but whether more intensive meditation practice in interventions will lead to better results in the long term remains unclear.

In addition to meditation practice, another important component in intervention is the disclosure or didactic component during courses, which not only consists of meditation guidance but also covers relevant Buddhist philosophy or ideas, such as the relationship between oneself and others or why it is important to be compassionate to others. Unlike the meditation skills, which are similar across interventions, the component of disclosure can vary from intervention to intervention, and study reports often mentioned the basic topic of disclosure but omitted the details. Although further analysis on this part of intervention is not possible, the present meta-analysis found that the two non-RCT interventions without weekly courses (Sears and Kraus, 2009; May et al., 2011) showed relatively small effect sizes. The lack of weekly courses may confound other factors such as lower motivation, but such results still implied the importance of other components besides meditation in interventions. Kang et al. (2015) reported that the group that discussed the ideas of loving-kindness but did not practice LKM could also change their attitudes toward oneself, though not their attitudes toward others. Their study did not evaluate positive emotions, but Neff and Germer (2013) found that the change in happiness was mediated by self-compassion, that is, kind attitudes toward oneself (Neff, 2003). Such findings implied that some effects do not come from meditation at all and highlighted the importance of the weekly courses in the interventions.

#### Different Types of Positive Emotions

Some researchers have noted that Buddhism endorses peaceful emotions rather than excitement (Koopmann-Holm et al., 2013; Lee et al., 2013) and that LKM also cultivates peaceful positive emotions (Kearney et al., 2014). Zeng et al. (2013) also noted that interpersonal kindness is an important quality of the positive emotions induced by LKM. In the present review, three studies distinguished the nature of positive emotions and supported that LKM mainly cultivates peaceful (Kearney et al., 2014) or pro-social positive emotions (Seppala et al., 2014; Hutcherson et al., 2015). In addition, Koopmann-Holm et al. (2013) also reported that LKM was able to enhance ideal (though not real) LAP emotions but not HAP emotions. Because the number of studies is small and the measurements varied across studies, the present meta-analysis cannot group studies according to the types of positive emotions and make meaningful comparisons. Notably, the results might be under-estimated if some measurements did not catch the special types of positive emotions cultivated by LKM or if some measurements were not sensitive to the change in emotions.

#### Individual Differences

Among the reviewed studies, only three studies evaluated LKM among samples with various clinical disorders or problems (Shahar et al., 2014, for high self-criticism; Johnson et al., 2011, for schizophrenia; Kearney et al., 2014, for PTSD). All of them reported significant increases in positive emotions, and excluding these studies did not change the results of the meta-analysis. Because of the limited number of studies, no special conclusion can be drawn about the influence of the clinical samples and more studies are required to evaluate the applicability of LKM in clinical populations.

In addition, some studies noted potential individual differences in interventions. Kok et al. (2013) reported that those participants with high positive emotions before intervention experienced sharper increases in positive emotions during the intervention. Cohn and Fredrickson (2010) also found that participants with high positive emotions are more likely to maintain meditation practice after the intervention. The high dropout rate in the study of Weytens et al. (2014) also implied that not all people are suitable for a meditation intervention. All of these findings were *post hoc* findings, and studies in the future can explore other potential individual variables.

### Implication for Future Studies and Practice

As discussed above, more studies are required to evaluate the effect of LKM interventions and on-going LKM. In addition to the basic effects, the present meta-analytic review also provides several theoretical implications for future studies.

First, what part of LKM interventions is the active component? Current protocols of LKM consist of many components. Previous studies on interventions evaluated the global effect of whole interventions, and studies in the future should try to identify which parts of the interventions are the active components. This not only illustrates the mechanism underlying the change but also benefits the optimization of the protocols, especially considering the time consumption of meditation-based intervention. As discussed above, the present meta-analytic review found that more meditation practice did not benefit the effect on positive emotions, but some evidence implied that didactic components may play important roles. Beyond positive emotions, previous studies on LKM paid more attention to meditation or considered LKM to be repeated mental practice but did not attach enough importance to the philosophy or ideas accompanying LKM. Until now, only a few qualitative studies mentioned that participants adopted the philosophy of LKM (e.g., Sears et al., 2011; Reddy et al., 2013). Such changes in beliefs might have long lasting influences on one’s life, and studies in the future should evaluate whether these changes in beliefs explain the effect of LKM interventions.

Second, is there a difference between the different psychological operations of LKM? The four immeasurables are conceptually different in terms of Buddhism, but whether the four subtypes of meditation have different effects was unknown. The systematic literature research in the present review found no empirical study that compared meditation on loving-kindness and meditation on compassion, and no study specifically focused on meditation that cultivated appreciative joy or equanimity regardless of positive emotions or other outcome variables. The present meta-analytic review is the first to identify and explain the potential different effects between these subtypes of LKM. Similarly, the targets of imagination in LKM are varied, and their order can be different depending on the traditions or interventions. As discussed above, no study compared the effects of blessing different targets on positive emotions, and the systematic literature search identified only three studies that involved such comparisons with other outcomes (Weng et al., 2013; Parks et al., 2014; Hutcherson et al., 2015). In summary, the potential differences between different psychological operations were not well evaluated, and more studies are required.

Third, what is the role of positive emotions in the effect of LKM interventions on other outcomes? As discussed above, recent studies have illustrated that LKM cultivated positive emotions with special natures, and this has an important theoretical implication. Among the reviewed studies, four studies followed the framework of the broaden-and-build theory of positive emotions (Fredrickson, 2001) and supported the idea that positive emotions played the mediator role between LKM interventions and other results such as well-being or interpersonal relationships (Fredrickson et al., 2008; Kok et al., 2013; Kearney et al., 2014; Schutte, 2014). However, the motivational dimensional model (Gable and Harmon-Jones, 2010) emphasized that the effects of positive emotions on attention are decided by motivation rather than valance, and Kearney et al. (2014) noted that their data were also compatible with the motivational dimensional model because they found that LKM cultivated unactivated positive emotions. Similarly, because the positive emotions cultivated by LKM have pro-social or other-focused characteristics (Zeng et al., 2013; Seppala et al., 2014), this pro-social characteristic may play a more essential role in the effect of LKM, especially on interpersonal relationships. In summary, future studies can further compare the role of (positive) emotions with (low) motivation and (pro-social) attitudes or beliefs.

In addition to the above theoretical implications, the findings in the present review also have implications for practice. First, the discussion on the self-selection problem and individual differences illustrated that LKM interventions are not suitable for everyone. In practice, it is necessary to clarify the requirement of meditation so that participants can decide whether they want to participate in the interventions. Second, different cultures have different understandings of “happiness,” and Eastern cultures prefer more peaceful emotions than Western cultures (see Koopmann-Holm et al., 2013; Lee et al., 2013). Therefore, if LKM mainly cultivated peaceful positive emotions, it may not match the expected “happiness” for some people. In summary, LKM is a promising intervention to promote long-term happiness, as Fredrickson et al. (2008) noted, but it is worthwhile to note the limitations of LKM in practice.

### Limitation of the Present Review

The following limitations should be noted. First, the present review only included studies published in peer-reviewed journals. However, considering that no publication bias was found, the risk that studies with non-significant results were not included in current review should be low. Second, because of the limited number of studies and availability of data, subgroup analysis, meta-regressions and comparisons with few studies may lack precision. In particular, the several aspects of variation across studies were evaluated independently, that is, other aspects of variation were not controlled when evaluating certain aspects of variation. Third, because details of the interventions were not available, the category of interventions into loving-kindness or compassion might be over-simplified. In summary, currently, the studies on LKM still represent the beginning stages of the research, and therefore, the conclusion of the present meta-analysis should also be considered as rather exploratory. Nevertheless, these exploratory findings note directions for future studies, and solid conclusions can be drawn with more rigorous and detailed studies.

## Conclusion

The present meta-analytic review confirmed that LKM interventions could enhance positive emotions in daily life and that the on-going practice of LKM could provide short-term positive emotions. Further analysis implied that (1) interventions focused on loving-kindness were more effective than interventions focused on compassion; and (2) didactic components were necessary while more intensive meditation did not enhance the effect. However, the mechanisms of LKM on positive emotions are still unclear, and potential limitations of applicability among people with different backgrounds should be considered in practice.

## Author Contributions

XZ, TO, and FL designed the study. XZ and CC reviewed articles, XZ, CC, and RW analyzed the data. XZ and TO wrote the paper. All authors discussed the results and commented on the manuscript.

## Conflict of Interest Statement

The authors declare that the research was conducted in the absence of any commercial or financial relationships that could be construed as a potential conflict of interest.
