# Supplementary material for: The effect of loving-kindness meditation on positive emotions: a meta-analytic review
Source: Front Psychol. 2015 Nov 3;6:1693. doi: 10.3389/fpsyg.2015.01693 (PMC4630307; doi:10.3389/fpsyg.2015.01693)
Supplement: Supplementary file 1 [file Data_Sheet_1.DOCX]

Supplementary Material

The effect of loving-kindness meditation on positive emotions: a meta-analytic review

Xianglong ZENG*, Cleo P. K. CHIU, Rong WANG, Tian P. S. OEI, Freedom Y. K. LEUNG

*** Correspondence:** Xianglong ZENG: psychologyzeng@gmail.com

# Supplementary Figures and Tables

## Supplementary Figures


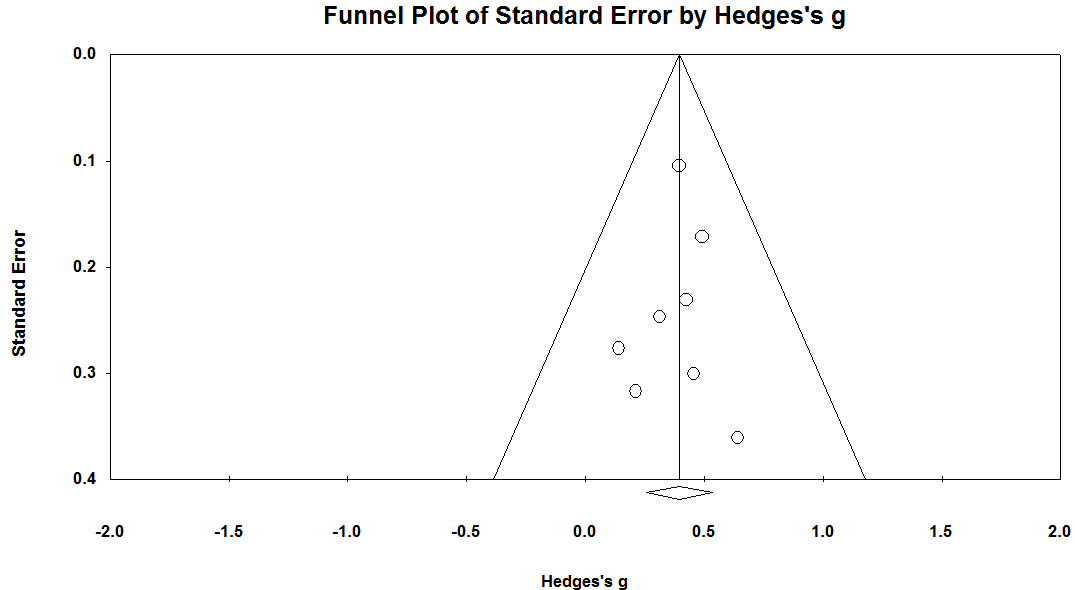


**Supplementary Figure 1. Funnel plot for RCT studies on daily PE, comparison with waitlist control group, based on highest estimation.**


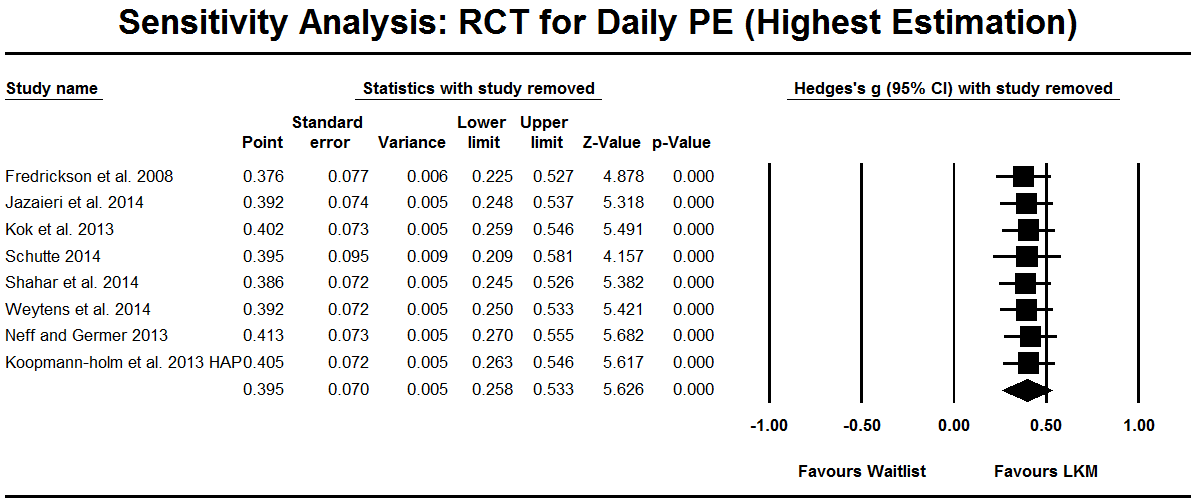


**Supplementary Figure 2. Sensitivity analysis with one study excluded for RCT studies on daily PE, comparison with waitlist control group. The highest estimation was based on high arousal positive emotion (HAP) in Koopmann-holm et al. (2013).**


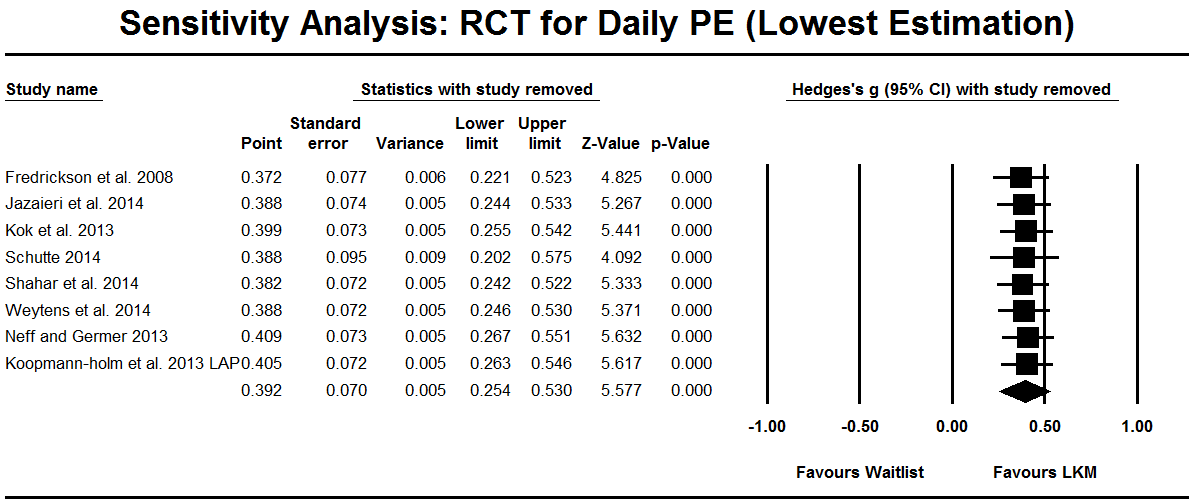


**Supplementary Figure 3. Sensitivity analysis with one study excluded for RCT studies on daily PE, comparison with waitlist control group. The lowest estimation was based on low arousal positive emotion (LAP) in Koopmann-holm et al. (2013).**

**
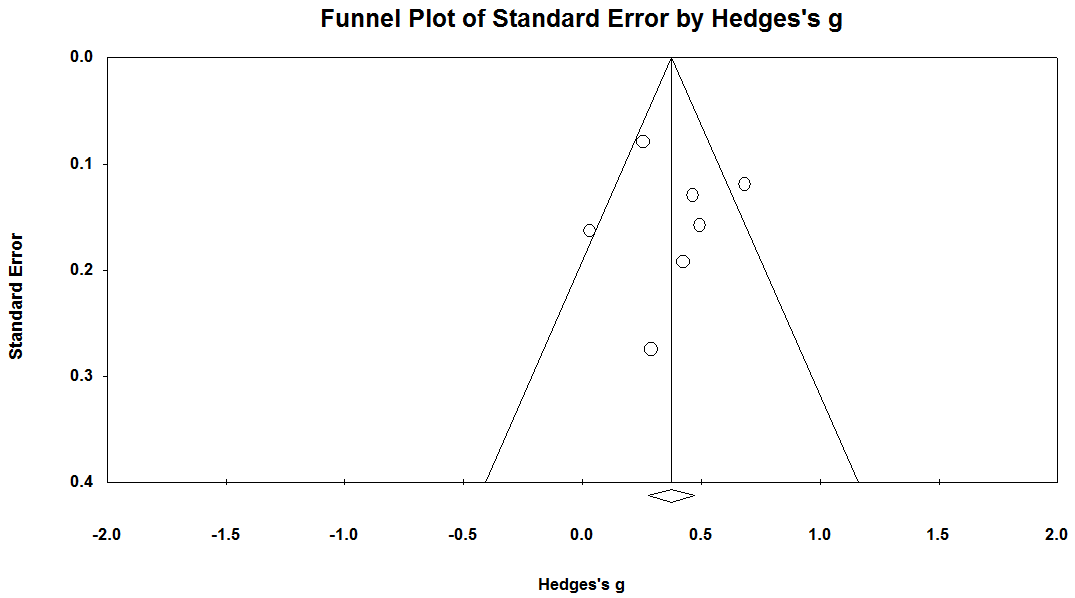
**

**Supplementary Figure 4. Funnel plot for non-RCT studies on daily PE, based on highest estimation.**


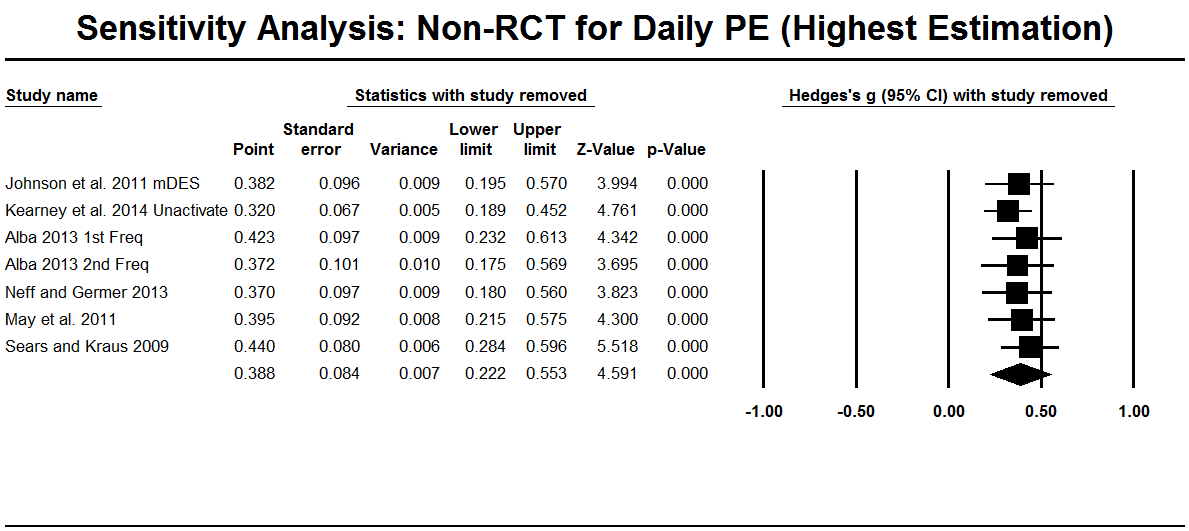


**Supplementary Figure 5. Sensitivity analysis with one study excluded for non-RCT studies on daily PE. The highest estimation was based on frequency of happiness (Freq) in Alba (2013), unactivated positive emotion (Unactivate) in Kearney et al. (2014) and modified Differential Emotions Scale (mDES) in Johnson et al. (2011). Subgroup analysis compared LKM with course and without course.**


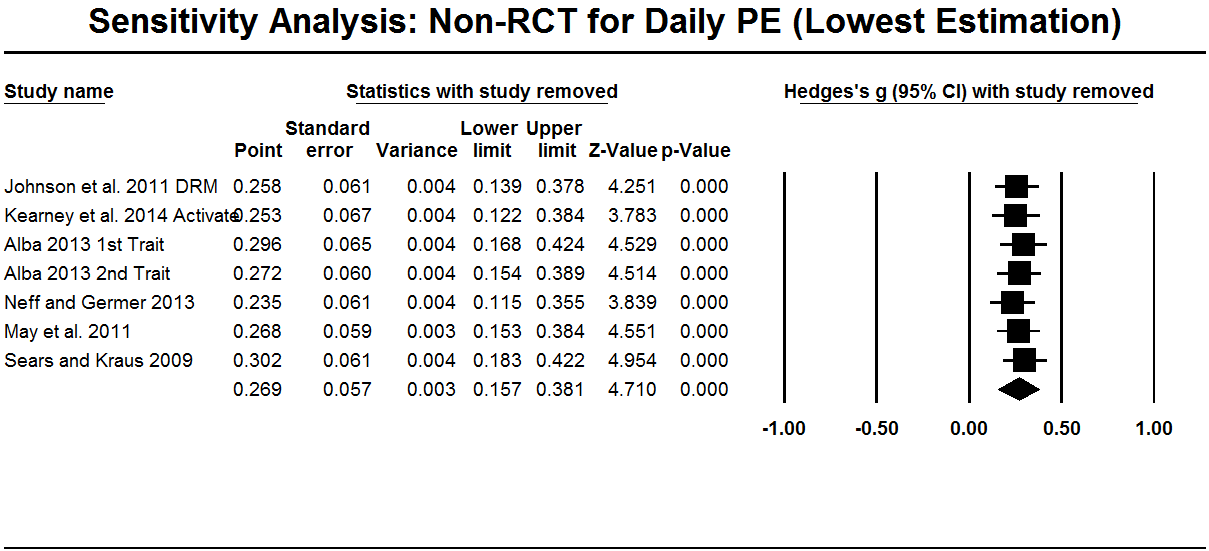


**Supplementary Figure 6. Sensitivity analysis with one study excluded for non-RCT studies on daily PE. The lowest estimation was based on long-term happiness (Trait) in Alba (2013), activated positive emotion (Activate) in Kearney et al. (2014), Day Reconstruction Method (DRM) in Johnson et al. (2011). Subgroup analysis compared LKM with course and without course.**

## Supplementary Tables

**Supplementary Table 1. Excluded studies and reasons.**

| Reason | Studies |
| --- | --- |
| Empirical studies on LKM without self-report positive emotions | Arch, Brown, Dean, Landy, Brown & Laudenslager (2014).  Barnhofer, Chittka, Nightingale, Visser & Crane (2010).  Boellinghaus, Jones & Hutton (2013).  Brewer, Worhunsky, Gray, Tang, Weber & Kober (2011).  Burgard, M. (2010).  Carson, Keefe, Lynch, Carson, Fras & Thorp (2005).  Condon, Desbordes, Miller & DeSteno (2013).  Crane, Jandric, Barnhofer & Williams (2010).  Desbordes, Negi, Pace, Wallace, Raison, & Schwartz (2012).  Garrison, Scheinost, Constable & Brewer (2014).  Hoge et al. (2013).  Hunsinger, Livingston, & Isbell (2013).  Hunsinger, Livingston, & Isbell (2014).  Jazaieri et al. (2013)  Johansson, Bjuhr & Ronnback (2014).  Johnson, Penn, Fredrickson, Meyer, Kring & Brantley (2009).  Kang, Gray & Dovidio (2014a).  Kang, Gray & Dovidio (2014b).  Kearney, Malte, McManus, Martinez, Felleman & Simpson (2013).  Kemper, Powell, Helms, & Kim-Shapiro (2015).  Kemper & Shaltout (2011).  Leung, Chan, Yin, Lee, So & Lee (2013).  Logie & Frewen (2014).  Lumma, Kok & Singer (2015).  Lutz, Lutz, Greischar, Rawlings & Ricard (2004).  Lutz, Brefczynski-Lewis, Johnstone & Davidson (2008).  Lutz, Greischar, Perlman & Davidson (2009).  Mascaro, Rilling, Negi & Raison (2013a).  Mascaro, Rilling, Negi & Raison (2013b).  McCall, Steinbeis, Ricard & Singer. (2014).  Pace et al. (2009)  Pace, Negi, Sivilli, Issa, Cole, Adame & Raison (2010).  Pace et al. (2013)  Parks, Birtel & Crisp (2014).  Reddy et al., (2013)  Sears, Kraus, Carlough & Treat (2011)  Shaltout, Tooze, Rosenberger & Kemper (2012).  Tonelli & Wachholtz (2014).  Wallmark, Safarzadeh, Daukantaite & Maddux (2013).  Weng et al. (2013)  Williams et al. (2005)  Xu et al. (2014) |
| Empirical Study not relevant to LKM | Albertson, Neff & Dill-Shackleford (2015).  Allen et al. (2012)  Arimitsu & Hofmann (2015).  Bach & Guse (2015).  Baer, Lykins & Peters (2012).  Bond, Mason, Lemaster, Shaw, Mullin, Holick & Saper (2013).  Carson (2006). Loving-kindness meditation findings not related to baseline differences.  Collinge, Kahn & Soltysik (2012).  Colzato, Hommel, van den Wildenberg & Hsieh (2010).  Colzato, Zech, Hommel, Verdonschot, van den Wildenberg & Hsieh (2012).  Danucalov et al. (2013)  Denny & Stevens (2011).  Engstrom & Soderfeldt (2010).  Frewen, Rogers, Flodrowski & Lanius (2015)  Greeson, Juberg, Maytan, James & Rogers (2014).  Hindman, Glass, Arnkoff & Maron (2014)  Hinton, Pich, Hofmann & Otto (2013).  Jennings, Frank, Snowberg, Coccia & Greenberg (2013).  Judge, Cleghorn, McEwan & Gilbert (2012).  Kelly, Zuroff, Foa & Gilbert (2010).  Kemeny et al. (2012).  Kemper, Bulla, Krueger, Ott, McCool & Gardiner (2011).  Kemper, Shaltout, Tooze & Rosenberger (2012).  Kim, Lee, Kim, Whang & Kang (2013).  Kjellgren & Taylor (2008).  Kozasa, Lacerda, Menezes, Wallace, Radvany, Mello & Sato (2015).  Kraus & Sears (2009).  Levenson, Ekman & Ricard (2012).  Lincoln, Hohenhaus & Hartmann (2013).  Lo (2014).  Lord (2013).  Lucre & Corten (2013).  Luders, Kurth, Mayer, Toga, Narr & Gaser (2012).  Mantzios, & Wilson (2014).  Moss, Wintering, Roggenkamp, Khalsa, Waldman, Monti & Newberg (2012).  Neff & Pommier (2013).  O'Connor, Berry, Stiver, & Rangan (2012).  Pace (2013).  Pidgeon, Ford & Klaassen (2014).  Pruitt & McCollum (2010). |
| Not empirical study that published on peer-reviewed journal | Alawabdeh & Salem (2015).  Berger (2011)  Desbordes, Negi, Pace, Wallace, Raison & Schwartz (2014).  Hinton, Ojserkis, Jalal, Peou & Hofmann (2013).  Kemper & Shaltout (2012).  Law (2012).  Negi, Pace, Wallace, Raison & Schwartz (2014).  Pace et al. (2012)  Ruchelli, Chapin, Darnall, Seppala, Doty & Mackey (2014). |

**Supplementary Table 2. Risk-of-bias assessment***

| **Study (main publication)** | **Sequence generation** | **Allocation concealment** | **Blinding of participant, staff and outcome** | **Incomplete outcome data** | **Selective reporting** | **Other sources of bias** |
| --- | --- | --- | --- | --- | --- | --- |
| Alba (2013) study 1 | High Risk | High Risk | High risk/ High risk/ High risk | High risk | High risk | Unclear risk |
|  | Not RCT study | Not RCT study | Nature of intervention made blinding impossible. Self-report limited emotions. | Parts of people agreed to answer questionnaire and there was further attribution. | Data for some non-significant measurement was mentioned but not specified. | No ITT analysis. |
| Alba (2013) study 2 | High Risk | High Risk | High risk/ High risk/High risk | High risk | High risk | Unclear risk |
|  | Not RCT study | Not RCT study | Nature of intervention made blinding impossible. Self-report limited emotions. | Parts of people agreed to answer questionnaire and there was further attribution. | Data for some non-significant measurement was mentioned but not specified. | No ITT analysis. |
| Feldman, et al., 2010 | High risk | High risk | Unclear risk/Unclear Risk/High Risk | Low risk | Low risk | Low risk |
|  | Allocation based on existed participants in groups. | Participants attending to a session would be assigned to the same condition. | Purpose of study in recruitment was not reported. No or very low previous experience of meditation. Unknown blind of staff. Self-report limited emotions. | No missing outcome data. | All listed outcomes reported, procedure of study mentioned clearly. | ITT analysis. Baseline difference unknown. |
| Fredrickson et al., 2008; Cohn & Fredrickson, 2010 | Unclear risk | Unclear risk | High risk/High risk/High risk | Low risk | Unclear risk | High risk |
|  | Randomized but no details reported. | Not specified | Nature of intervention made blinding impossible. Self-report limited emotions. | Attribution was not significantly different across groups in numbers or characteristics. | All listed outcomes reported, but whole procedure was not clear. | ITT analysis conducted but statistic unknown, and result is different from completers. No baseline difference between conditions (experiential condition was not significant in model). |
| Huncherson et al., (2008) | Unclear risk | Unclear risk | Unclear risk /Unclear risk /High risk | Low risk | Low risk | Unknown risk |
|  | Randomized but no details reported. | Not specified. | Purpose of study in recruitment was not report. No or very low previous experience of meditation. Unknown blind of staff. Self-report limited emotions. | No missing data. | All listed outcomes reported, procedure of study mentioned clearly. | ITT Analysis, Baseline difference unknown. |
| Hutcherson et al. (2015) | High risk | High risk | Low risk /Unclear Risk/High Risk | Low risk | High risk | Low risk |
|  | Not RCT study. | Not RCT study.. | Cover the purpose of meditation study to participants. No or very low previous experience of meditation. Unknown blind of staff. Self-report limited emotions. | One participant among 19 and additional 3 participants were excluded because of technical difficulties. | Some non-interested emotions (e.g. afraid, surprised) were not further reported. | No other problems identified. |
| Jazaieri et al. (2014) | Low risk | Unclear risk | High risk/High risk/High risk | Low risk | Unclear risk | Unclear risk |
|  | Random number generator used(A) | Not specified. | Nature of intervention made blinding impossible. Self-report limited emotions. | “No significant difference…in the percentage of participants who dropped after being randomly assigned to CCT (n = 9; 15 %) and WL (n = 10; 25 %). | All listed outcomes reported, but whole procedure was not clear. | No ITT result reported. Baseline difference between group was unknown. |
| Johnson et al. (2011) | High Risk | High Risk | High risk /High risk/High risk | Low Risk | Unclear Risk | Low Risk |
|  | Not RCT study | Not RCT study | Nature of intervention made blinding impossible. Self-report limited emotions. | No attribution pre- to post-test, 2 lost in follow-up. | All listed outcomes reported, but whole procedure was not clear. | ITT Analysis |
| Kearney et al. (2014) | High risk | High risk | High risk /High risk/High risk | Low risk | Unclear risk | Low risk |
|  | Not RCT study | Not RCT study | Nature of intervention made blinding impossible. Self-report limited emotions. | 42 ITT to 37 completers and 34 3-month follow-up. | All listed outcomes reported, but whole procedure was not clear. | ITT analysis (last observation forward). |
| Klimecki et al., (2013) | Unclear risk | Low risk | High risk/High risk/High risk | Low risk | Unknown risk | High risk |
|  | Randomized but no details reported. | “Allocation of participants to training groups was performed without revealing the specific content of the study…” | Nature of intervention made blinding impossible. Self-report limited emotions. | “Five participants were excluded due to unreturned pre-training questionnaire data or due to technical problems during the pre-training fMRI measurement” | Numbers of experiments were conducted and whether all results were reported was unknown. | Based on completers, and no baseline data available. |
| Klimecki et al., (2014) | Unclear risk | Unclear risk | High risk/High risk/High risk | Low risk | Unknown risk | High risk |
|  | Randomized but no details reported. | “Allocation of participants to training groups was performed without revealing the specific content of the study…” | Nature of intervention made blinding impossible. Self-report limited emotions. | “included age and depression scores as covariates in the analyses.” | Numbers of experiments were conducted and whether all results were reported was unknown. | Based on completers, and no baseline data available. |
| Kok et al. (2013) | Unknown risk | Unknown risk | High risk/High risk/High risk | Low risk | Unclear risk | Low risk |
|  | Randomized but no details reported. | Not specified | Nature of intervention made blinding impossible. Self-report limited emotions. | “6 were ultimately excluded, 5 for failure to attend the meditation workshops and 1 for previous meditation experience” | All listed outcomes reported, but whole procedure was not clear. | ITT analysis conducted although method unknown, and no difference from completers. “neither experimental condition…significantly predicted baseline positive emotions scores” |
| Koopmann-Holm et al. (2013) | Low risk | Low risk | High risk/ High risk/ High risk | Low risk | Unclear risk | High risk |
|  | Random number generator used(A) | The project manager did not know the content of class.(A) | Nature of intervention made blinding impossible. Self-report limited emotions. | “There were no significant differences in the percentage of participants who dropped out by condition.” | All listed outcomes reported, but whole procedure was not clear. | No ITT analysis.  Baseline difference unknown. |
| Leiberg et al. (2011) | Unclear risk | Unclear risk | High risk/ High risk/High risk | Unclear risk | Low risk | Unclear risk |
|  | Randomized but no details reported. | “Allocation of participants to training groups was performed without revealing the specific content of the study…” | Nature of intervention made blinding impossible. Self-report limited emotions. | Attribution was not significant different from completers. | Many experiments were conducted and whether all results were reported was unknown. | No ITT analysis.  Baseline difference unknown. |
| May et al. (2011) | High risk | High risk | High risk /High risk/High risk | Low risk | Unclear risk | Low risk |
|  | Not RCT study | Not RCT study | Nature of intervention made blinding impossible. Self-report limited emotions. | No attribution | All listed outcomes reported, but whole procedure was not clear. | ITT analysis. |
| May et al. (2014) | Unclear risk | Unclear risk | High risk/ High risk/ High risk | Low risk | Unclear risk | Unclear risk |
|  | Randomized but no details reported. | Not specified. | Nature of intervention made blinding impossible. Self-report limited emotions. | One participant dropped in each group. | All listed outcomes reported, but whole procedure was not clear. | No ITT analysis. "those in the LKM condition started the study with higher positive affect". |
| Neff and Germer (2013) study 1 | High risk | High risk | High risk/ High risk/High risk | Unclear risk | Unclear risk | Unclear risk |
|  | Not RCT study | Not RCT study | Nature of intervention made blinding impossible. Self-report limited emotions. | 23 ITT to 21 completers | All listed outcomes reported, but whole procedure was not clear. | No ITT analysis. |
| Neff & Germer (2013) study 2 | Low risk | High risk | High risk/ High risk/ High risk | High risk | Unclear risk | Unclear risk |
|  | Random number generator used(A) | No strategy in place to conceal random allocations.(A) | Nature of intervention made blinding impossible. Self-report limited emotions. | Dropout (3 participants) happened in LKM group only. | All listed outcomes reported, but whole procedure was not clear. | No ITT analysis.  No group difference on baseline measurements. |
| Schutte (2014) | Unclear risk | Unclear risk | High risk/ High risk/ High risk | Low risk | Unclear risk | Low risk |
|  | Randomized but no details reported. | Not specified | Nature of intervention made blinding impossible. Self-report limited emotions. | “There was no significant difference in number of participants  completing the post-test in the intervention condition  (200) and the control condition (174)” | All listed outcomes reported, but whole procedure was not clear. | ITT conducted (last observation forward) and the result was not essentially changed.  No group difference on baseline measurements. |
| Sears and Kraus (2009) | High risk | High risk | High risk/ /Low risk/High risk | Low risk | Unclear risk | Unclear risk |
|  | Not RCT study | Not RCT study | Not blinding to participants but blinding to instructors. Self-report limited emotions. | 17 out of 20 students participated intervention group | All listed outcomes reported, but whole procedure was not clear. | ITT analysis. |
| Seppala et al., (2015) | Unclear risk | Unclear risk | Unclear risk/Unclear risk/High risk | Low risk | Low risk | Low risk |
|  | Randomized but no details reported. | Not specified. | Cover the purpose of meditation study to participants. Unknown previous experience of meditation. Unknown blind of staff. Self-report limited emotions. | No missing data. | All listed outcomes reported, procedure of study mentioned clearly. | ITT Analysis, “The findings remained significant when controlling for any group differences in baseline”. |
| Shahar et al., (2014) | Unclear risk | Unclear risk | High risk/ High risk/ High risk | High risk | Unclear risk | Low risk |
|  | Randomized but no details reported. | Not specified | Nature of intervention made blinding impossible. Self-report limited emotions. | Four of 5 dropout in intervention group due to less attendance of course. Only 1 in wait list control group dropped at the same period. | All listed outcomes reported, but whole procedure was not clear. | “The ITT analyses were identical to the PP analyses when psychological distress variables were examined.” No group difference on baseline measurements. |
| Weytens et al., (2014) | Unclear risk | Unclear risk | High risk/ High risk/ High risk | High risk | Unclear risk | Low risk |
|  | Randomized but no details reported. | Not specified | Nature of intervention made blinding impossible. Self-report limited emotions. | 43% dropout in LKM group, significantly higher than other group of 11%, partially due to participants’ dislikeness to meditation. | All listed outcomes reported, but whole procedure was not clear. | No ITT analysis.  No group difference on baseline measurements. |
| Wheeler et al. (2015) | Unclear risk | Unclear risk | Unclear risk /Unclear risk /High risk | Low risk | Low risk | High risk |
|  | Randomized but no details reported. | Not Specified | Purpose of study in recruitment was not reported. Unknown blind of staff. Self-report limited emotions. | No missing data | All listed outcomes reported, procedure of study mentioned clearly. | ITT analysis. Baseline difference between groups was larger than pre-post difference in groups. |

* The study by Lee et al. (2012) was not included because it was based on cross-sectional design.

(A) Information adopted from supplemental material of Galante et al. (2014) with permission, which was gained through their communication with authors.

**Supplementary Table 3. Summary of outcomes for RCT studies that evaluated the effect of intervention on daily positive emotions**

| Study | Comparison | Outcome | Data Used for Evaluation | Significance (Preference) in Original Article | Hedge’s g (Standard Error) |
| --- | --- | --- | --- | --- | --- |
| Fredrickson et al. (2008); Cohn & Fredrickson (2010) | Wait List | Modified Differential Emotions Scale – positive emotions | LKM: M-Pre = 2.634, SD-Pre = 0.607, M-Post = 2.829, SD-Post = 0.693, N = 67.  Ctrl: M-Pre = 2.798, SD-Pre = 0.715, M-Post = 2.657, SD-Post = 0.667, N = 72 (A) | S(LKM): “The fixed effect for experimental condition and week were not significant, but their interaction was” | 0.492 (0.171) |
| Jazaieri et al. (2014) | Wait List | Subjective Happiness Scale | LKM: M-Pre = 16.76, SD-Pre = 3.20, M-Post = 17.41, SD-Post = 3.05, N = 50.  Ctrl: M-Pre = 17.04, SD-Pre = 2.40, M-Post = 16.46, SD-Post = 2.52, N = 30 | S(LKM): F test (2 groups × 2 times) showed LKM group had larger increase in PE than ctrl group. | 0.425(0.231) |
| Kok et al. (2013) | Wait List | Modified Differential Emotions Scale – positive emotions | LKM: M-Pre = 2.854, SD-Pre = 0.667, M-Post = 3.025, SD-Post = 0.826, N = 31.  Ctrl: M-Pre = 2.867, SD-Pre = 0.787, M-Post = 2.769, SD-Post = 0.867, N = 34 | S(LKM): “experimental condition significantly predicted slope of change in positive emotions” | 0.313(0.247) |
| Koopmann-Holm et al. (2013) | Mindfulness Meditation | Affect Valuation Index – actual high arousal positive emotions | LKM: M-Pre = 2.765, SD-Pre = 0.752, M-Post = 2.961, SD-Post = 0.949, N = 17.  Ctrl: M-Pre = 2.632, SD-Pre = 0.793, M-Post = 2.649, SD-Post = 0.789, N = 19 (A) | NS (B): F test (ANCOVA with baseline real emotions and post-intervention ideal emotions controlled) did not find difference in post-intervention real emotions between meditations groups (combined LKM and mindfulness meditation) and non-meditation groups (theater therapy and no intervention). | 0.202(0.327)(B) |
|  | Mindfulness Meditation | Affect Valuation Index – actual low arousal positive emotions | LKM: M-Pre = 2.784, SD-Pre = 0.857, M-Post = 3.177, SD-Post = 0.818, N = 17.  Ctrl: M-Pre = 2.877, SD-Pre = 0.747, M-Post = 3.101, SD-Post = 0.861, N = 19. (A) | NS (B): F test (ANCOVA with baseline real emotions and post-intervention ideal emotions controlled) did not find difference in post-intervention real emotions between meditations groups (combined LKM and mindfulness meditation) and non-meditation groups (theater therapy and no intervention). | 0.196(0.327)(B) |
|  | Theater Therapy | Affect Valuation Index – actual high arousal positive emotions | LKM: M-Pre = 2.784, SD-Pre = 0.857, M-Post = 3.177, SD-Post = 0.818, N = 17.  Ctrl: M-Pre = 2.375, SD-Pre = 0.642, M-Post = 2.563, SD-Post = 0.451, N = 16. (A) | NS (B): F test (ANCOVA with baseline real emotions and post-intervention ideal emotions controlled) did not find difference in post-intervention real emotions between meditations groups (combined LKM and mindfulness meditation) and non-meditation groups (theater therapy and no intervention). | 0.010(0.340) (B) |
|  | Theater Therapy | Affect Valuation Index – actual low arousal positive emotions | LKM: M-Pre = 2.765, SD-Pre = 0.752, M-Post = 2.961, SD-Post = 0.949, N = 17.  Ctrl: M-Pre = 2.792, SD-Pre = 0.815, M-Post = 2.917, SD-Post = 0.735, N = 16. (A) | NS (B): F test (ANCOVA with baseline real emotions and post-intervention ideal emotions controlled) did not find difference in post-intervention real emotions between meditations groups (combined LKM and mindfulness meditation) and non-meditation groups (theater therapy and no intervention). | 0.336(0.342) (B) |
|  | Wait List | Affect Valuation Index – actual high arousal positive emotions | LKM: M-Pre = 2.765, SD-Pre = 0.752, M-Post = 2.961, SD-Post = 0.949, N = 17.  Ctrl: M-Pre = 2.288, SD-Pre = 0.486, M-Post = 2.318, SD-Post = 0.586, N = 22. (A) | NS (B): F test (ANCOVA with baseline real emotions and post-intervention ideal emotions controlled) did not find difference in post-intervention real emotions between meditations groups (combined LKM and mindfulness meditation) and non-meditation groups (theater therapy and no intervention). | 0.213(0.317) (B) |
|  | Wait List | Affect Valuation Index – actual low arousal positive emotions | LKM: M-Pre = 2.784, SD-Pre = 0.857, M-Post = 3.177, SD-Post = 0.818, N = 17.  Ctrl: M-Pre = 2.455, SD-Pre = 0.767, M-Post = 2.742, SD-Post = 0.642, N = 22. (A) | NS (B): F test (ANCOVA with baseline real emotions and post-intervention ideal emotions controlled) did not find difference in post-intervention real emotions between meditations groups (combined LKM and mindfulness meditation) and non-meditation groups (theater therapy and no intervention). | 0.144(0.317)(B) |
| Leiberg et al. (2011) | Matched memory training | PANAS (unknown time) – positive affect | F = 3.86 (A)  LKM: N = 27  Ctrl: N = 32 | NS (B): F test (2 groups × 2 times) did not found significant interaction. | 0.507(0.262) (B) |
| May et al. (2014) | Matched concentration meditation | PANAS (past few days) – positive affects | Not Available  LKM: N = 15  Ctrl: N = 14 | S (LKM): “LKM model revealed a significant positive slope during the meditation period”, which showed LKM group had higher increase in PE than Ctrl group. |  |
| Neff & Germer (2013) study 2 | Wait list | Subjective Happiness Scale | LKM: M-Pre = 3.86, SD-Pre = 1.20, M-Post = 4.38, SD-Post = 1.14, N = 24.  Ctrl: M-Pre = 3.46, SD-Pre = 1.47, M-Post = 3.78, SD-Post = 1.58, N = 27. | NS: F test (2 groups × 2 times) did not find significant interaction. | 0.142(0.277) |
| Schutte (2014) | Wait list | PANAS (past month) – positive affects | LKM: M-Pre = 37.55, SD-Pre = 6.73, M-Post = 37.59, SD-Post = 8.50, N = 200.  Ctrl: M-Pre = 36.88, SD-Pre = 6.26, M-Post = 33.66, SD-Post = 7.88, N = 174. | S(LKM): F test (ANCOVA with pre interventions controlled) found LKM had larger increase in PE than Ctrl. | 0.396(0.104) |
| Shahar et al., (2014) | Wait list | PANAS (last week) – positive affects | LKM: M-Pre = 2.76, SD-Pre = 0.86, M-Post = 3.27, SD-Post = 1.11, N = 14.  Ctrl: M-Pre = 2.93, SD-Pre = 0.84, M-Post = 2.81, SD-Post = 0.81, N = 17. | S(LKM): LKM group showed significant increase in PE in pre-post t test, but Ctrl group did not. | 0.642(0.361) |
| Weytens et al., (2014) | Positive Emotion Regulation | Subjective Happiness Scale | LKM: M-Pre = 4.76, SD-Pre = 1.44, M-Post = 5.27, SD-Post = 1.13, N = 16.  Ctrl: M-Pre = 4.51, SD-Pre = 1.31, M-Post = 5.13, SD-Post = 1.16, N = 27. | Not calculated in original article. | 0.094(0.310) |
|  | Wait list | Subjective Happiness Scale | LKM: M-Pre = 4.76, SD-Pre = 1.44, M-Post = 5.27, SD-Post = 1.13, N = 16.  Ctrl: M-Pre = 4.48, SD-Pre = 1.58, M-Post = 4.37, SD-Post = 1.42, N = 35. | Not calculated in original article. | 0.456(0.301) |

(A) Data was collected by personal communication with authors.

(B) The significance was reported based on original article where ideal emotions were controlled and two meditations groups and other two groups were combined together. However the effect size was evaluated without controlling ideal emotions.

**Supplementary Table 4. Summary of outcomes for non-RCT studies that evaluated the effect of intervention on daily positive emotions**

| Study | Outcome | Data Used for Evaluation | Significance in Original Article* | Hedge’s g (Standard Error)* |
| --- | --- | --- | --- | --- |
| Alba (2013) study 1 | Fordyce Emotions Questionnaire–long-term happiness | Pre-M = 7.20, Pre-SD = 1.01, Post-M = 7.50, Post-SD = 1.54, r = .851, N = 20 | NS | 0.182(0.118) |
|  | Fordyce Emotions Questionnaire–frequency of happiness | Pre-M = 44.00, Pre-SD = 18.25, Post-M = 49.75, Post-SD = 21.37, r = .934, N = 20 | S | 0.256(0.079) |
| Alba (2013) study 2 | Fordyce Emotions Questionnaire–long-term happiness | Pre-M = 6.75, Pre-SD = 1.76, Post-M = 7.20, Post-SD = 1.84, r = .377, N = 31 | NS | 0.244(0.198) |
|  | Fordyce Emotions Questionnaire–frequency of happiness | Pre-M = 44.77, Pre-SD = 21.13, Post-M = 55.87, Post-SD = 24.51, r = .754, N = 31 | S | 0.465(0.130) |
| Johnson et al. (2011) | Modified Differential Emotions Scale (past 2 weeks) – positive emotions | Pre-M = 1.50, Pre-SD = 0.59, Post-M = 1.78, Post-SD = 0.66, r = .667, N = 18 | S | 0.425(0.193) |
|  | Day Reconstruction Method (certain day) – positive emotions | Pre-M = 2.05, Pre-SD = 0.50, Post-M = 2.37, Post-SD = 0.88, r = .766, N = 16 | S | 0.351(0.168) |
| Kearney et al. (2014) | Circumplex Measure of Emotion (past 7 days) – unactivated positive emotions | Pre-M = 6.6, Pre-SD = 3.1, Post-M = 9.4, Post-SD = 4.3, N = 42 (A) | S | 0.683(0.119) |
|  | Circumplex Measure of Emotion (past 7 days) – activated positive emotions | Pre-M = 9.6, Pre-SD = 5.5, Post-M = 11.4, Post-SD = 5.8, N = 42 (A) | NS | 0.312(0.110) |
| May et al. (2011) | PANAS (unknown time) – positive affect | t = -1.080, N = 12 | NS | 0.290(0.275) |
| Neff and Germer (2013) study 1 | Subjective Happiness Scale | Pre-M = 3.83, Pre-SD = 1.50, Post-M = 4.57, Post-SD = 1.36, N = 21 (A) | S | 0.494(0.158) |
| Sears and Kraus (2009) | PANAS (past week) – positive affect | Pre-M = 33.53, Pre-SD = 5.8, Post-M = 33.32, Post-SD = 6.49, N = 17 (A) | NS | 0.032(0.163) |

* All data was based on single group pre-post t test.

(A) The correlations between pre- post-measurement were not available and set to .75, this value in other studies were provided by authors through personal communication.

**Supplementary Table 5. Summary of outcomes for RCT studies that evaluated effect of on-going practice of LKM on immediate positive emotions**

| Study | Comparison | Outcome | Data Used for Evaluation | Significance (Preference) in Original Article | Hedge’s g (Standard Error) |
| --- | --- | --- | --- | --- | --- |
| Huncherson et al., (2008) | Neutral visualization | Positive affects (combined calm, happy, loving) | F = 11.17  LKM: N = 45  Ctrl: N = 48 | S (LKM): F test (2 groups × 2 times) showed LKM group had larger increase in PE than Ctrl group. | 0.688 (0.212) |
| Seppala et al., (2015) | Neutral visualization | Self-focused positive emotions (proud, self-esteem, self-satisfaction) | LKM: M-Pre = 3.77, M-Post = 3.72, t = 0.48, N = 46  Ctrl: M-Pre = 4.08, M-Post = 4.00, t = 0.63, N = 44 | NS: Both groups did not show significant change in pre-post t test. | 0.038 (0.209) |
|  | Neutral visualization | Other-focused positive emotions (friendly, close to others, affection, loving) | LKM: M-Pre = 4.00, M-Post = 4.41, t = -3.83, N = 46  Ctrl: M-Pre = 4.61, M-Post = 4.86, t = 0.85, N = 44 | S (LKM): LKM group showed significant increase in PE in pre-post t test, but Ctrl group did not. | 0.109 (0.209) |
|  | Induction of Pride | Self-focused positive emotions (proud, self-esteem, self-satisfaction) | LKM: M-Pre = 3.77, M-Post = 3.72, t = 0.48, N = 46  Ctrl: M-Pre = 3.95, M-Post = 4.55, t = -4.04, N = 44 | S (Ctrl): Ctrl group showed significant increase in PE in pre-post t test, but LKM group did not. | -0.755 (0.217) |
|  | Induction of Pride | Other-focused positive emotions (friendly, close to others, affection, loving) | LKM: M-Pre = 4.00, M-Post = 4.41, t = -3.83, N = 46  Ctrl: M-Pre = 4.61, M-Post = 4.86, t = -1.93, N = 44 | S (LKM): LKM group showed significant increase in PE in pre-post t test, but Ctrl group did not. | 0.200 (0.210) |
| Klimecki et al., (2013) | Memory skill | Positive affect when viewing suffering in video (single item) | t = 2.72 (post intervention independent t test) (B)  LKM: N = 28  Ctrl: N = 30 | S (LKM): post intervention independent t test showed LKM group had significant higher PE than Ctrl group. | 0.705 (0.267) |
| Klimecki et al., (2014) | Memory skill | Positive affect when viewing suffering in video (single item) | t = 4.37 (post intervention independent t test) (B)  LKM: N = 25  Ctrl: N = 28 | S (LKM): post intervention independent t test showed LKM group had significant higher PE than Ctrl group. | 1.185 (0.295) |
| Wheeler et al. (2015) | Music | PANAS (current moment) - positive affects | LKM: M-Pre = 30.13, SD-Pre = 7.02, M-Post = 28.63, SD-Post = 9.47, N = 32  Ctrl: M-Pre = 26.40, SD-Pre = 6.70, M-Post = 24.77, SD-Post = 8.40, N = 30 | Not calculated in original article. | 0.014 (0.251) |
| Feldman et al. (2010) | Mindfulness Breath | PANAS (“right now”) - positive affects | Not Available  LKM: N = 59  Ctrl: N = 68 | S(LKM): not reported in original article. (A) | Not Available |
|  | Progressive Muscle | PANAS (“right now”) - positive affects | Not Available  LKM: N = 59  Ctrl: N = 63 | NS: not reported in original article. (A) | Not Available |

* S = significant, NS = not significant, PE = positive emotions, LKM = Loving-kindness meditation, Ctrl = control condition.

(A) The data for this study was not provided by authors and the information was provided by Galante et al. (2014).

(B) Only post-intervention tasks involved active application of LKM, thereby independent t test at post intervention was used.

**Supplementary Table 6. Summary of outcomes for studies with other design**

| Study | Design | Outcome | Data Used for Evaluation | Significance in original article | Hedge’s g (Standard Error)* |
| --- | --- | --- | --- | --- | --- |
| Hutcherson et al. (2015) | Within subject design, on-going practice.  LKM versus Neutral imagination | Social connection positive emotions (friendly, loving, happy, joyful) | t = 4.68, N = 18 (within group t-test) | S: LKM condition showed significant higher PE in within group t-test. | 1.054(0.286) |
|  | Within subject design, on-going practice.  LKM versus Neutral imagination | Self-focused positive emotions (self-esteem, being proud) | t = 1.98, N = 18 (within group t-test) | NS: no significant difference between two conditions in within group t-test. | 0.446(0.237) |
| Lee et al. (2012) | Cross-sectional comparison between meditators and novices, daily positive emotions | Chinese Affect Scale – positive affect | Meditators: M = 23.5, SD = 5.6, N = 11  Novices, M = 23.1, SD = 5.5, N = 11 | NS: No significant difference was found in between group t test. | 0.069(0.410) |

# References

Alawabdeh, E., Salem. (2015). Compassion in mental health.*Middle East Journal of Nursing, 9*(1), 21-24.

Alba, B. (2013). Loving-kindness meditation: A field study.*Contemporary Buddhism, 14*(2), 187-203. doi:10.1080/14639947.2013.832494

Albertson, E. R., Neff, K. D., & DillShackleford, K. E. (2015). Self-compassion and body dissatisfaction in women: A randomized controlled trial of a brief meditation intervention.*Mindfulness, 6*(3), 444. doi: 10.1007/s12671-014-0277-3

Allen, M., Dietz, M., Blair, K. S., van Beek, M., Rees, G., Vestergaard-Poulsen, P., . . . Roepstorff, A. (2012). Cognitive-affective neural plasticity following active-controlled mindfulness intervention.*Journal of Neuroscience,32*(44), 15601-15610. doi:10.1523/jneurosci.2957-12.2012

Arch, J. J., Brown, K. W., Dean, D. J., Landy, L. N., Brown, K. D., & Laudenslager, M. L. (2014). Self-compassion training modulates alpha-amylase, heart rate variability, and subjective responses to social evaluative threat in women.*Psychoneuroendocrinology, 42*, 49-58. doi:10.1016/j.psyneuen.2013.12.018

Arimitsu, K., & Hofmann, S. G. (2015). Cognitions as mediators in the relationship between self-compassion and affect.*Personality and Individual Differences, 74*, 41-48.

Bach, J., M., & Guse, T. (2015). The effect of contemplation and meditation on ‘great compassion’ on the psychological well-being of adolescents.*Journal of Positive Psychology, 10*(4), 359-369. doi:10.1080/17439760.2014.965268

Baer, R. A., Lykins, E. L., & Peters, J. R. (2012). Mindfulness and self-compassion as predictors of psychological wellbeing in long-term meditators and matched nonmeditators.*The Journal of Positive Psychology, 7*(3), 230-238.

Barnhofer, T., Chittka, T., Nightingale, H., Visser, C., & Crane, C. (2010). State effects of two forms of meditation on prefrontal EEG asymmetry in previously depressed individuals.*Mindfulness, 1*(1), 21-27.

Berger, R. E. (2011). Re: Effect of compassion meditation on neuroendocrine, innate immune and behavioral responses to psychosocial stress.*Journal of Urology, 186*(4), 1325-1326.

Boellinghaus, I., Jones, F. W., & Hutton, J. (2013). Cultivating self-care and compassion in psychological therapists in training: The experience of practicing loving-kindness meditation.*Training and Education in Professional Psychology, 7*(4), 267-277.

Bond, A. R., Mason, H. F., Lemaster, C. M., Shaw, S. E., Mullin, C. S., Holick, E. A., & Saper, R. B. (2013). Embodied health: The effects of a mind-body course for medical students.*Medical Education Online, 18*, 1-8.

Brewer, J. A., Worhunsky, P. D., Gray, J. R., Tang, Y. Y., Weber, J., & Kober, H. (2011). Meditation experience is associated with differences in default mode network activity and connectivity.*Proceedings of the National Academy of Sciences of the United States of America, 108*(50), 20254-9.

Burgard, M. (2010). The effect of positive affect induction via metta meditation on the attentional blink.*Journal of Articles in Support of the Null Hypothesis, 7*(1), 8.

Carson, J. W. (2006). Loving-kindness meditation findings not related to baseline differences.*Journal of Holistic Nursing, 24*(1), 5-6.

Carson, J., Keefe, F., Lynch, T., Carson, K., V, G., Fras, A., & Thorp, S. (2005). Loving-kindness meditation for chronic low back pain: Results from a pilot trial.*J Holist Nurs, 23*(3), 287-304.

Cohn, M. A., & Fredrickson, B. L. (2010). In search of durable positive psychology interventions: Predictors and consequences of long-term positive behavior change.*The Journal of Positive Psychology, 5*(5), 355-366.

Collinge, W., Kahn, J., & Soltysik, R. (2012). Promoting reintegration of national guard veterans and their partners using a self-directed program of integrative therapies: A pilot study.*Military Medicine, 177*(12), 1477-85.

Colzato, L. S., Hommel, B., van den Wildenberg, W. P., & Hsieh, S. (2010). Buddha as an eye opener: A link between prosocial attitude and attentional control.*Frontiers in Psychology, 1*, 156.

Colzato, L. S., Zech, H., Hommel, B., Verdonschot, R., van den Wildenberg, W. P., & Hsieh, S. (2012). Loving-kindness brings loving-kindness: The impact of buddhism on cognitive self-other integration.*Psychonomic Bulletin & Review, 19*(3), 541-5.

Condon, P., Desbordes, G., Miller, W. B., & DeSteno, D. (2013). Meditation increases compassionate responses to suffering.*Psychological Science, 24*(10), 2125-2127. doi: 10.1177/0956797613485603

Crane, C., Jandric, D., Barnhofer, T., & Williams, J. (2010). Dispositional mindfulness, meditation, and conditional goal setting.*Mindfulness, 1*(4), 204-214.

Danucalov, M. A. D., Kozasa, E. H., Ribas, K. T., Galduroz, J. C. F., Garcia, M. C., Verreschi, I. T. N., . . . Leite, J. R. (2013). A yoga and compassion meditation program reduces stress in familial caregivers of alzheimer's disease patients.*Evidence-Based Complementary and Alternative Medicine,*, 8. doi:10.1155/2013/513149

Denny, J., & Stevens, L. (2011). EEG/loreta frequency and localization characteristics of compassion versus egocentrism versus universal mind.*Journal of Neurotherapy, 15 (4)*, 434.

Desbordes, G., Negi, L. T., Pace, T. W. W., Wallace, B. A., Raison, C. L., & Schwartz, E. L. (2014). Effects of eight-week meditation training on hippocampal volume: A comparison of mindful attention training and cognitively-based compassion training.*Journal of Alternative and Complementary Medicine, 20 (5)*, A24.

Desbordes, G., Negi, L. T., Pace, T. W., Wallace, B., Raison, C. L., & Schwartz, E. L. (2012). Effects of mindful-attention and compassion mediation training on amygdala response to emotional stimuli in an ordinary, non-meditative state.*Frontiers in Human Neuroscience, 6*, 292.

Engstrom, M., & Soderfeldt, B. (2010). Brain activation during compassion meditation: A case study.*Journal of Alternative & Complementary Medicine, 16*(5), 597-9.

Feldman, G., Greeson, J., & Senville, J. (2010). Differential effects of mindful breathing, progressive muscle relaxation, and loving-kindness meditation on decentering and negative reactions to repetitive thoughts.*Behaviour Research & Therapy, 48*(10), 1002-11.

Fredrickson, B. L., Cohn, M. A., Coffey, K. A., Pek, J., & Finkel, S. M. (2008). Open hearts build lives: Positive emotions, induced through loving-kindness meditation, build consequential personal resources.*Journal of Personality & Social Psychology, 95*(5), 1045-62.

Frewen, P., Rogers, N., Flodrowski, L., & Lanius, R. (2015). Mindfulness and metta-based trauma therapy (mmtt): Initial development and proof-of-concept of an internet resource.*Mindfulness,*doi: 10.1007/s12671-015-0402-y

Galante, J., Galante, I., Bekkers, M. J., & Gallacher, J. (2014). Effect of kindness-based meditation on health and well-being: A systematic review and meta-analysis. Journal of consulting and clinical psychology, 82(6), 1101.

Garrison, K. A., Scheinost, D., Constable, R. T., & Brewer, J. A. (2014). BOLD signal and functional connectivity associated with loving kindness meditation.*Brain and Behavior, 4*(3), 337-47.

Greeson, J. M., Juberg, M. K., Maytan, M., James, K., & Rogers, H. (2014). A randomized controlled trial of koru: A mindfulness program for college students and other emerging adults.*Journal of American College Health,62*(4), 222-233.

Hindman, R. K., Glass, C. R., Arnkoff, D. B., & Maron, D. D. (2014). A comparison of formal and informal mindfulness programs for stress reduction in university students.*Mindfulness Aug,*(Pagination), No Pagination Specified.

Hinton, D. E., Pich, V., Hofmann, S. G., & Otto, M. W. (2013). Acceptance and mindfulness techniques as applied to refugee and ethnic minority populations with PTSD: Examples from "culturally adapted CBT".*Cognitive and Behavioral Practice, 20*(1), 33-46.

Hinton, D. E., Ojserkis, R. A., Jalal, B., Peou, S., & Hofmann, S. G. (2013). Loving-kindness in the treatment of traumatized refugees and minority groups: A typology of mindfulness and the nodal network model of affect and affect regulation.*Journal of Clinical Psychology, 69*(8), 817-828.

Hoge, E. A., Chen, M. M., Orr, E., Metcalf, C. A., Fischer, L. E., Pollack, M. H., . . . Simon, N. M. (2013). Loving-kindness meditation practice associated with longer telomeres in women.*Brain, Behavior, & Immunity, 32*, 159-63.

Hunsinger, M., Livingston, R., & Isbell, L. (2013). The impact of loving-kindness meditation on affective learning and cognitive control.*Mindfulness, 4*(3), 275-280.

Hunsinger, M., Livingston, R., & Isbell, L. (2014). Spirituality and intergroup harmony: Meditation and racial prejudice.*Mindfulness, 5*(2), 139-144.

Hutcherson, C. A., Seppala, E. M., & Gross, J. J. (2008). Loving-kindness meditation increases social connectedness.*Emotion, 8*(5), 720-4.

Hutcherson, C. A., Seppala, E. M., & Gross, J. J. (2015). The neural correlates of social connection.*Cognitive Affective & Behavioral Neuroscience, 15*(1), 1-14. doi:10.3758/s13415-014-0304-9

Jazaieri, H., Jinpa, G. T., McGonigal, K., Rosenberg, E. L., Finkelstein, J., Simon-Thomas, E., . . . Goldin, P. R. (2013). Enhancing compassion: A randomized controlled trial of a compassion cultivation training program.*Journal of Happiness Studies, 14*(4), 1113-1126.

Jazaieri, H., McGonigal, K., Jinpa, T., Doty, J. R., Gross, J. J., & Goldin, P. R. (2014). A randomized controlled trial of compassion cultivation training: Effects on mindfulness, affect, and emotion regulation.*Motivation and Emotion, 38*(1), 23-35.

Jennings, P. A., Frank, J. L., Snowberg, K. E., Coccia, M. A., & Greenberg, M. T. (2013). Improving classroom learning environments by cultivating awareness and resilience in education (CARE): Results of a randomized controlled trial.*School Psychology Quarterly, 28*(4), 374-390. doi:10.1037/spq0000035

Johansson, B., Bjuhr, H., & Ronnback, L. (2014). Evaluation of an advanced mindfulness programme following a mindfulness-based stress reduction programme for participants suffering from mental fatigue after acquired brain injury.*Brain Injury, 28 (5-6)*, 573.

Johnson, D. P., Penn, D. L., Fredrickson, B. L., Kring, A. M., Meyer, P. S., Catalino, L. I., & Brantley, M. (2011). A pilot study of loving-kindness meditation for the negative symptoms of schizophrenia.*Schizophrenia Research,129*(2-3), 137-40.

Johnson, D. P., Penn, D. L., Fredrickson, B. L., Meyer, P. S., Kring, A. M., & Brantley, M. (2009). Loving-kindness meditation to enhance recovery from negative symptoms of schizophrenia.*Journal of Clinical Psychology,65*(5), 499-509.

Judge, L., Cleghorn, A., McEwan, K., & Gilbert, P. (2012). An exploration of group-based compassion focused therapy for a heterogeneous range of clients presenting to a community mental health team.*International Journal of Cognitive Therapy, 5*(4), 420-429.

Kang, Y., Gray, J. R., & Dovidio, J. F. (2014). The head and the heart: Effects of understanding and experiencing lovingkindness on attitudes toward the self and others.*Mindfulness Oct,*(Pagination), No Pagination Specified.

Kang, Y., Gray, J. R., & Dovidio, J. F. (2014). The nondiscriminating heart: Lovingkindness meditation training decreases implicit intergroup bias.*Journal of Experimental Psychology: General, 143*(3), 1306-1313.

Kearney, D. J., Malte, C. A., McManus, C., Martinez, M. E., Felleman, B., & Simpson, T. L. (2013). Loving-kindness meditation for posttraumatic stress disorder: A pilot study.*Journal of Traumatic Stress, 26*(4), 426-34.

Kearney, D. J., McManus, C., Malte, C. A., Martinez, M. E., Felleman, B., & Simpson, T. L. (2014). Loving-kindness meditation and the broaden-and-build theory of positive emotions among veterans with posttraumatic stress disorder.*Medical Care, 52 Suppl 5*, S32-8.

Kelly, A. C., Zuroff, D. C., Foa, C. L., & Gilbert, P. (2010). Who benefits from training in self-compassionate self-regulation? A study of smoking reduction.*Journal of Social and Clinical Psychology, 29*(7), 727-755.

Kemeny, M. E., Foltz, C., Cavanagh, J. F., Cullen, M., Giese-Davis, J., Jennings, P., . . . Ekman, P. (2012). Contemplative/emotion training reduces negative emotional behavior and promotes prosocial responses.*Emotion,12*(2), 338-50.

Kemper, K., Bulla, S., Krueger, D., Ott, M. J., McCool, J. A., & Gardiner, P. (2011). Nurses' experiences, expectations, and preferences for mind-body practices to reduce stress.*BMC Complementary & Alternative Medicine, 11*, 26.

Kemper, K., & Shaltout, H. (2012). Non-verbal communication of compassion: Feasibility of measuring psychophysiological effects of blind exposure.*BMC Complementary and Alternative Medicine, 12*

Kemper, K., Shaltout, H., Tooze, J., & Rosenberger, E. (2012). Time, touch, and compassion: Effects on autonomic nervous system and well-being.*BMC Complementary and Alternative Medicine, 12*

Kemper, K. J., Powell, D., Helms, C. C., & Kim-Shapiro, D. B. (2015). Loving-kindness meditation's effects on nitric oxide and perceived well-being: A pilot study in experienced and inexperienced meditators.*Explore: The Journal of Science and Healing, 11*(1), 32-39. doi: 10.1016/j.explore.2014.10.002

Kemper, K. J., & Shaltout, H. A. (2011). Non-verbal communication of compassion: Measuring psychophysiologic effects.*BMC Complementary & Alternative Medicine, 11*, 132.

Kim, D. K., Lee, K. M., Kim, J., Whang, M. C., & Kang, S. W. (2013). Dynamic correlations between heart and brain rhythm during autogenic meditation.*Frontiers in Human Neuroscience, 7*, 8. doi:10.3389/fnhum.2013.00414

Kjellgren, A., & Taylor, S. (2008). Mapping zazen meditation as a developmental process: Exploring the experiences of experienced and inexperienced meditators.*Journal of Transpersonal Psychology, 40*(2), 224-250.

Klimecki, O. M., Leiberg, S., Lamm, C., & Singer, T. (2013). Functional neural plasticity and associated changes in positive affect after compassion training.*Cerebral Cortex, 23*(7), 1552-1561. doi:10.1093/cercor/bhs142

Klimecki, O. M., Leiberg, S., Ricard, M., & Singer, T. (2014). Differential pattern of functional brain plasticity after compassion and empathy training.*Social Cognitive and Affective Neuroscience, 9*(6), 873-879. doi:10.1093/scan/nst060

Kok, B. E., Coffey, K. A., Cohn, M. A., Catalino, L. I., Vacharkulksemsuk, T., Algoe, S. B., . . . Fredrickson, B. L. (2013). How positive emotions build physical health: Perceived positive social connections account for the upward spiral between positive emotions and vagal tone.*Psychological Science, 24*(7), 1123-32.

Koopmann-Holm, B., Sze, J., Ochs, C., & Tsai, J. L. (2013). Buddhist-inspired meditation increases the value of calm.*Emotion, 13*(3), 497-505. doi:10.1037/a0031070

Kozasa, E. H., Lacerda, S. S., Menezes, C., Wallace, B. A., Radvany, J., Mello, L. E. A. M., & Sato, J. R. (2015). Effects of a 9-day shamatha buddhist meditation retreat on attention, mindfulness and self-compassion in participants with a broad range of meditation experience.*Mindfulness,*doi: dx.doi.org/10.1007/s12671-015-0385-8

Kraus, S., & Sears, S. (2009). Measuring the immeasurables: Development and initial validation of the self-other four immeasurables (SOFI) scale based on buddhist teachings on loving kindness, compassion, joy, and equanimity.*Social Indicators Research, 92*(1), 169-181.

Law, R. W. (2012). An analogue study of loving-kindness meditation as a buffer against social stress.*Dissertation Abstracts International: Section b: The Sciences and Engineering, 72*(7-B), 4365.

Lee, T. M., Leung, M. K., Hou, W. K., Tang, J. C., Yin, J., So, K. F., . . . Chan, C. C. (2012). Distinct neural activity associated with focused-attention meditation and loving-kindness meditation.*PLoS ONE [Electronic Resource], 7*(8), e40054.

Leiberg, S., Klimecki, O., & Singer, T. (2011). Short-term compassion training increases prosocial behavior in a newly developed prosocial game.*Plos One, 6*(3), 10. doi:10.1371/journal.pone.0017798

Leung, M. K., Chan, C. C., Yin, J., Lee, C. F., So, K. F., & Lee, T. M. (2013). Increased gray matter volume in the right angular and posterior parahippocampal gyri in loving-kindness meditators.*Social Cognitive & Affective Neuroscience, 8*(1), 34-9.

Levenson, R. W., Ekman, P., & Ricard, M. (2012). Meditation and the startle response: A case study.*Emotion, 12*(3), 650-658. doi:10.1037/a0027472

Lincoln, T. M., Hohenhaus, F., & Hartmann, M. (2013). Can paranoid thoughts be reduced by targeting negative emotions and self-esteem? an experimental investigation of a brief compassion-focused intervention.*Cognitive Therapy and Research, 37*(2), 390-402. doi:10.1007/s10608-012-9470-7

Lo, H. H. (2014). Applications of buddhist compassion practices among people suffering from depression and anxiety in confucian societies in east asia.*Journal of Religion & Spirituality in Social Work: Social Thought, 33*(1), 19-32.

Logie, K., & Frewen, P. (2014). Self/other referential processing following mindfulness and loving-kindness meditation.*Mindfulness Jul,*(Pagination), No Pagination Specified.

Lord, S. A. (2013). Meditative dialogue: Cultivating compassion and empathy with survivors of complex childhood trauma.*Journal of Aggression, Maltreatment & Trauma, 22*(9), 997-1014.

Lucre, K. M., & Corten, N. (2013). An exploration of group compassion-focused therapy for personality disorder.*Psychology and Psychotherapy-Theory Research and Practice, 86*(4), 387-400. doi:10.1111/j.2044-8341.2012.02068.x

Luders, E., Kurth, F., Mayer, E. A., Toga, A. W., Narr, K. L., & Gaser, C. (2012). The unique brainan atomy of meditation practitioners: Alterations in cortical gyrification.*Frontiers in Human Neuroscience, 6*, 9. doi:10.3389/fnhum.2012.00034

Lumma, A. -., Kok, B. E., & Singer, T. (2015). Is meditation always relaxing? investigating heart rate, heart rate variability, experienced effort and likeability during training of three types of meditation.*International Journal of Psychophysiology, 97*(1), 38-45. doi: 10.1016/j.ijpsycho.2015.04.017

Lutz, A., Lutz, L. L., Greischar, N. B., Rawlings, M., & Ricard, R. J. (2004). Long-term meditators self-induce high-amplitude gamma synchrony during mental practice.*Proceedings of the National Academy of Sciences of the United States of America, 101*(46), 16369-16373. doi:10.1073/pnas.0407401101

Lutz, A., Brefczynski-Lewis, J., Johnstone, T., & Davidson, R. J. (2008). Regulation of the neural circuitry of emotion by compassion meditation: Effects of meditative expertise.*PLoS ONE [Electronic Resource], 3*(3), e1897.

Lutz, A., Greischar, L. L., Perlman, D. M., & Davidson, R. J. (2009). BOLD signal in insula is differentially related to cardiac function during compassion meditation in experts vs. novices.*Neuroimage, 47*(3), 1038-46.

Mantzios, M., & Wilson, J. C. (2014). Exploring mindfulness and mindfulness with self-compassion-centered interventions to assist weight loss: Theoretical considerations and preliminary results of a randomized pilot study.*Mindfulness Jul,*(Pagination), No Pagination Specified.

Mascaro, J. S., Rilling, J. K., Negi, L. T., & Raison, C. L. (2013). Pre-existing brain function predicts subsequent practice of mindfulness and compassion meditation.*Neuroimage, 69*, 35-42.

Mascaro, J. S., Rilling, J. K., Tenzin Negi, L., & Raison, C. L. (2013). Compassion meditation enhances empathic accuracy and related neural activity.*Social Cognitive & Affective Neuroscience, 8*(1), 48-55.

May, C. J., Burgard, M., Mena, M., Abbasi, I., Bernhardt, N., Clemens, S., . . . Williamson, R. (2011). Short-term training in loving-kindness meditation produces a state, but not a trait, alteration of attention.*Mindfulness,2*(3), 143-153.

May, C. J., Weyker, J. R., Spengel, S. K., Finkler, L. J., & Hendrix, S. E. (2014). Tracking longitudinal changes in affect and mindfulness caused by concentration and loving-kindness meditation with hierarchical linear modeling.*Mindfulness, 5*(3), 249-258.

McCall, C., Steinbeis, N., Ricard, M., & Singer, T. (2014). Compassion meditators show less anger, less punishment, and more compensation of victims in response to fairness violations.*Frontiers in Behavioral Neuroscience,8*, 424.

Moss, A. S., Wintering, N., Roggenkamp, H., Khalsa, D. S., Waldman, M. R., Monti, D., & Newberg, A. B. (2012). Effects of an 8-week meditation program on mood and anxiety in patients with memory loss.*Journal of Alternative and Complementary Medicine, 18*(1), 48-53. doi:10.1089/acm.2011.0051

Neff, K. D., & Germer, C. K. (2013). A pilot study and randomized controlled trial of the mindful self-compassion program.*Journal of Clinical Psychology, 69*(1), 28-44.

Neff, K. D., & Pommier, E. (2013). The relationship between self-compassion and other-focused concern among college undergraduates, community adults, and practicing meditators.*Self and Identity, 12*(2), 160-176.

Negi, L. T., Pace, T. W. W., Wallace Raison, C. L., & Schwartz, E. L. (2014). Effects of eight-week meditation training on hippocampal volume: A comparison of mindful attention training and cognitively-based compassion training.*Journal of Alternative and Complementary Medicine (New York, N.Y.), 20*(5), A24.

O'Connor, L. E., Berry, J. W., Stiver, D. J., & Rangan, R. K. (2012). Depression, guilt, and tibetan buddhism.*Psychology, 3*(9A), 805.

Pace, S. (2013). Does religion affect the materialism of consumers? an empirical investigation of buddhist ethics and the resistance of the self.*Journal of Business Ethics, 112*(1), 25-46.

Pace, T., Negi, L., Donaldson-Lavelle, B., Ozawa-de Silva, B., Reddy, S., Cole, S., . . . Raison, C. (2012). Cognitively-based compassion training reduces peripheral inflammation in adolescents in foster care with high rates of early life adversity.*BMC Complementary and Alternative Medicine, 12*

Pace, T. W., Negi, L. T., Adame, D. D., Cole, S. P., Sivilli, T. I., Brown, T. D., . . . Raison, C. L. (2009). Effect of compassion meditation on neuroendocrine, innate immune and behavioral responses to psychosocial stress.*Psychoneuroendocrinology, 34*(1), 87-98.

Pace, T. W., Negi, L. T., Sivilli, T. I., Issa, M. J., Cole, S. P., Adame, D. D., & Raison, C. L. (2010). Innate immune, neuroendocrine and behavioral responses to psychosocial stress do not predict subsequent compassion meditation practice time.*Psychoneuroendocrinology, 35*(2), 310-5.

Pace, T. W. W., Negi, L. T., Dodson-Lavelle, B., Ozawa-de Silva, B., Reddy, S. D., Cole, S. P., . . . Raison, C. L. (2013). Engagement with cognitively-based compassion training is associated with reduced salivary C-reactive protein from before to after training in foster care program adolescents.*Psychoneuroendocrinology, 38*(2), 294-299.

Parks, S., Birtel, M. D., & Crisp, R. J. (2014). Evidence that a brief meditation exercise can reduce prejudice toward homeless people.*Social Psychology, 45*(6), 458-465.

Pidgeon, A. M., Ford, L., & Klaassen, F. (2014). Evaluating the effectiveness of enhancing resilience in human service professionals using a retreat-based mindfulness with metta training program: A randomised control trial.*Psychology Health & Medicine, 19*(3), 355-364. doi:10.1080/13548506.2013.806815

Pruitt, I. T., & McCollum, E. E. (2010). Voices of experienced meditators: The impact of meditation practice on intimate relationships.*Contemporary Family Therapy: An International Journal, 32*(2), 135-154.

Reddy, S. D., Negi, L. T., Dodson-Lavelle, B., Ozawa-de Silva, B., Pace, T. W. W., Cole, S. P., . . . Craighead, L. W. (2013). Cognitive-based compassion training: A promising prevention strategy for at-risk adolescents.*Journal of Child and Family Studies, 22*(2), 219-230. doi:10.1007/s10826-012-9571-7

Ruchelli, G., Chapin, H., Darnall, B., Seppala, E., Doty, J., & Mackey, S. (2014). Compassion meditation training for people living with chronic pain and their significant others: A pilot study and mixed-methods analysis.*Journal of Pain, 1)*, S117.

Schutte, N. S. (2014). The broaden and build process: Positive affect, ratio of positive to negative affect and general self-efficacy.*Journal of Positive Psychology, 9*(1), 66-74. doi:10.1080/17439760.2013.841280

Sears, S., Kraus, K., Carlough, E., & Treat. (2011). Perceived benefits and doubts of participants in a weekly meditation study.*Mindfulness, 2*(3), 167-174. doi:10.1007/s12671-011-0055-4

Sears, S., & Kraus, S. (2009). I think therefore I om: Cognitive distortions and coping style as mediators for the effects of mindfulness meditation on anxiety, positive and negative affect, and hope.*Journal of Clinical Psychology, 65*(6), 561-73.

Seppala, E., Seppala, C., Hutcherson, D., Nguyen, J., Doty, J., & Gross. (2014). Loving-kindness meditation: A tool to improve healthcare provider compassion, resilience, and patient care.*Journal of Compassionate Health Care, 1*(1) doi:10.1186/s40639-014-0005-9

Shahar, B., Shahar, O., Szsepsenwol, S., Zilcha Mano, N., Haim, O., Zamir, S., . . . Levit-Binnun. (2014). A wait-list randomized controlled trial of loving-kindness meditation programme for self-criticism.*Clinical Psychology & Psychotherapy,*doi:10.1002/cpp.1893

Shaltout, H. A., Tooze, J. A., Rosenberger, E., & Kemper, K. J. (2012). Time, touch, and compassion: Effects on autonomic nervous system and well-being.*Explore: The Journal of Science & Healing, 8*(3), 177-84.

Tonelli, M. E., & Wachholtz, A. B. (2014). Meditation-based treatment yielding immediate relief for meditation-naive migraineurs.*Pain Management Nursing, 15*(1), 36-40.

Wallmark, E., Safarzadeh, K., Daukantaite, D., & Maddux, R. E. (2013). Promoting altruism through meditation: An 8-week randomized controlled pilot study.*Mindfulness, 4*(3), 223-234.

Weng, H. Y., Fox, A. S., Shackman, A. J., Stodola, D. E., Caldwell, J. Z. K., Olson, M. C., . . . Davidson, R. J. (2013). Compassion training alters altruism and neural responses to suffering.*Psychological Science, 24*(7), 1171-1180.

Weytens, F., Luminet, O., Verhofstadt, L. L., & Mikolajczak, M. (2014). An integrative theory-driven positive emotion regulation intervention.*PLoS ONE [Electronic Resource], 9*(4), e95677.

Wheeler, E. A. (2014). Brief compassion meditation and recall of positive-emotion words.*Journal of Articles in Support of the Null Hypothesis, 11*(2), 12.

Williams, A. L., Selwyn, P. A., Liberti, L., Molde, S., Njike, V. Y., McCorkle, R., . . . Katz, D. L. (2005). A randomized controlled trial of meditation and massage effects on quality of life in people with late-stage disease: A pilot study.*Journal of Palliative Medicine, 8*(5), 939-52.

Xu, J., Vik, A., Groote, I. R., Lagopoulos, J., Holen, A., Ellingsen, O., . . . Davanger, S. (2014). Nondirective meditation activates default mode network and areas associated with memory retrieval and emotional processing. *Frontiers in Human Neuroscience, 8*, 10. doi:10.3389/fnhum.2014.00086
